# Therapeutic Effect of Modulating TREM-1 via Anti-inflammation and Autophagy in Parkinson’s Disease

**DOI:** 10.3389/fnins.2019.00769

**Published:** 2019-08-02

**Authors:** Chien-Wei Feng, Nan-Fu Chen, Chun-Sung Sung, Hsiao-Mei Kuo, San-Nan Yang, Chien-Liang Chen, Han-Chun Hung, Bing-Hung Chen, Zhi-Hong Wen, Wu-Fu Chen

**Affiliations:** ^1^National Museum of Marine Biology & Aquarium, Pingtung, Taiwan; ^2^Department of Marine Biotechnology and Resources, National Sun Yat-sen University, Kaohsiung City, Taiwan; ^3^Division of Neurosurgery, Department of Surgery, Kaohsiung Armed Forces General Hospital, Kaohsiung City, Taiwan; ^4^Department of Neurological Surgery, Tri-Service General Hospital, National Defense Medical Center, Taipei, Taiwan; ^5^Department of Anesthesiology, Taipei Veterans General Hospital, Taipei, Taiwan; ^6^School of Medicine, National Yang-Ming University, Taipei, Taiwan; ^7^Center for Neuroscience, National Sun Yat-sen University, Kaohsiung City, Taiwan; ^8^Department of Pediatrics, E-Da Hospital, Kaohsiung City, Taiwan; ^9^School of Medicine, College of Medicine, I-Shou University, Kaohsiung City, Taiwan; ^10^Division of Nephrology, Kaohsiung Veterans General Hospital, Kaohsiung City, Taiwan; ^11^Department of Medicine, National Yang-Ming University, Taipei, Taiwan; ^12^Department of Biotechnology, Kaohsiung Medical University, Kaohsiung City, Taiwan; ^13^Department of Medical Research, Kaohsiung Medical University Hospital, Kaohsiung City, Taiwan; ^14^Institute of Biomedical Sciences, National Sun Yat-sen University, Kaohsiung City, Taiwan; ^15^Department of Neurosurgery, Kaohsiung Chang Gung Memorial Hospital and Chang Gung University College of Medicine, Kaohsiung City, Taiwan; ^16^Department of Neurosurgery, Xiamen Chang Gung Hospital, Xiamen, China

**Keywords:** TREM-1, Parkinson’s disease, neuroinflammation, autophagy, zebrafish, rat

## Abstract

Parkinson’s disease (PD) is one of the most common age-related neurodegenerative diseases, and neuroinflammation has been identified as one of its key pathological characteristics. Triggering receptors expressed on myeloid cells-1 (TREM-1) amplify the inflammatory response and play a role in sepsis and cancer. Recent studies have demonstrated that the attenuation of TREM-1 activity produces cytoprotective and anti-inflammatory effects in macrophages. However, no study has examined the role of TREM-1 in neurodegeneration. We showed that LP17, a synthetic peptide blocker of TREM-1, significantly inhibited the lipopolysaccharide (LPS)-induced upregulation of proinflammatory cascades of inducible nitric oxide synthase (iNOS), cyclooxygenase-2, and nuclear factor-kappa B. Moreover, LP17 enhanced the LPS-induced upregulation of autophagy-related proteins such as light chain-3 and histone deacetylase-6. We also knocked down TREM-1 expression in a BV2 cell model to further confirm the role of TREM-1. LP17 inhibited 6-hydroxydopamine-induced locomotor deficit and iNOS messenger RNA expression in zebrafish. We also observed therapeutic effects of LP17 administration in 6-hydroxydopamine-induced PD syndrome using a rat model. These data suggest that the attenuation of TREM-1 could ameliorate neuroinflammatory responses in PD and that this neuroprotective effect might occur via the activation of autophagy and anti-inflammatory pathways.

## Introduction

Neurodegenerative diseases (NDs) are permanent conditions of the central nervous system (CNS); in 2015, ND was ranked as the third leading cause of death in high-income countries, with 60 deaths per 10,000 people. Some recent studies depicted that among all causes of NDs, neuroinflammation played a crucial role, especially in NDs, such as Alzheimer’s disease (AD), Parkinson’s disease (PD), and Huntington’s disease (Kempuraj et al., 2016; Ransohoff, 2016). Neuroinflammation is primarily mediated by microglial cell over activation, which was suggested to enhance the release of inflammatory enzymes (Ransohoff, 2016; Villegas-Llerena et al., 2016).

In PD, which is one of the most common NDs, highly activated microglia have been found in the substantia nigra (SN) of patients’ brains (Koshimori et al., 2015). Recent studies showed that inhibition of lipopolysaccharide (LPS)-induced inflammatory responses, including the production of nitride oxide (NO) and prostaglandin E2 (PGE-2), might reduce PD symptoms more than clinical drugs can (Liberatore et al., 1999; Sairam et al., 2003; Teismann et al., 2003; Wallace and Del Soldato, 2003; Rocha et al., 2015; Tentillier et al., 2016). However, specific inhibition of downstream inflammatory mediators, such as NO and PGE-2, for clinical PD treatment, remains to have ambiguous effects or even had some severe side effects (Chen et al., 2000; Steinbach et al., 2000; Cho et al., 2004). Up to the present, only few studies have investigated the effect of modulation of the upstream inflammatory signaling [i.e., triggering receptor expressed on myeloid cells (TREM) family] in PD (Liu et al., 2016; Tan et al., 2016).

The TREM family is a member of the immunoglobulin superfamily. The receptor contains three parts, including an extracellular immunoglobulin domain, a transmembrane region, and a short tail in the cytoplasm (Colonna and Facchetti, 2003). It includes at least two receptors, the TREM-1 and TREM-2. In general, TREM-1 is mainly expressed by neutrophils, macrophages, and mature monocytes; whereas TREM-2 is mainly expressed by microglia and osteoclasts (Bouchon et al., 2000; Sharif and Knapp, 2008). TREM-1 is non-covalently associated with the DNAX activation protein of 12 kDa (DAP12). Phosphorylation of DAP12 leads to binding of the Src homology 2 (SH2) domains to form receptor complexes for further stimulation and amplification of the inflammatory response (Shen and Sigalov, 2017; Tammaro et al., 2017). Previous studies showed that TREM-1 played a key role in some diseases, such as inflammatory bowel disease, acute pancreatitis, gouty arthritis, and atherosclerosis. On the other hand, TREM-2 participates in inhibition of inflammatory cytokine production in microbial challenge and could help improve ND-related symptoms (Paloneva et al., 2002; Schmid et al., 2002; Gibot et al., 2006b; Kamei et al., 2010). However, the characteristics of TREM-2 remain controversial and further investigations on it as a drug target could be risky. In addition, no ligands for any of the TREM-2 receptor have been identified. In contrast, TREM-1 has been shown by all related studies to have consistent characteristics in peripheral inflammatory disease; this would be an advantage in future development.

A recent study demonstrated that expression of TREM-1 in mouse lungs was increased by tumor-specific knockout of autophagy-related gene (Guo et al., 2013). Autophagy is a lysosome-mediated degradation process that controls the quality of cytoplasmic components and organelles (Kroemer et al., 2010; Yang and Klionsky, 2010). It plays a critical role in pathogen elimination and cytokine production of macrophages (Singh et al., 2006; Saitoh et al., 2008b). Studies revealed that inhibition of autophagy in macrophages led to an increase in inflammatory factors, such as inducible nitric oxide synthase (iNOS), cyclooxygenase-2 (COX-2), and NO in immune cells (Plantinga et al., 2012; Matsuzawa-Ishimoto et al., 2018). The studies mentioned above proved that inhibition of autophagy could enhance the inflammatory response. The same phenomenon was observed in an AD brain, where autophagy could modulate Aβ-mediated activation of microglia. Moreover, knockdown of LC3 or ATG-7 was demonstrated to induce activation of the nucleotide-binding domain, leucine-rich repeat-containing, pyrin domain-containing 3 (NLRP3) inflammasome, and enhance interleukin-1 beta (IL-1β) secretion in cultured microglia treated with fibrillar Aβ (Cho et al., 2014). However, the effect of TREM-1-mediated modulation of autophagy on inflammation or neuroinflammation remains unknown. Therefore, we attempted to inhibit the activity of TREM-1 using LP17 in BV-2 microglia for further investigation.

A researcher synthesized LP17 to specifically antagonize TREM-1 receptor activity (Gibot et al., 2006a). Previous studies have likewise demonstrated successful LP17 inhibition of TREM-1 activity in lung injury, ischemia, and septic shock (Gibot et al., 2008; Pelham et al., 2014; Yuan et al., 2016). These researches assured the specificity and efficacy of LP17 on TREM-1 inhibition. In our research, we intended to manipulate the activity of TREM-1 using LP17 in a PD model and to investigate the role of TREM-1 in PD.

## Materials and Methods

### Reagents

Dulbecco’s modified Eagle’s medium/F12 medium, fetal bovine serum (FBS), sodium pyruvate, L-glutamine, antibiotic–antimycotic solution, and trypsin-EDTA were purchased from Invitrogen Co. (Grand Island, NY, United States). LPS (*Escherichia coli*), 6-hydroxydopamine (6-OHDA), dimethyl sulfoxide (DMSO), and bafilomycin A1 from *Streptomyces griseus* were purchased from Sigma Co., Ltd. (St. Louis, MO, United States). D-Methamphetamine were purchased from Taiwan Food and Drug Administration (TFDA). The TREM-1 blocking peptide LP17 (LQVTDSGLYRCVIYHPP) and the control peptide (TDSRCVIGLYHPPLQVY) were referred from previous study in a septic shock model (Gibot et al., 2004b). LP17 and the control peptide were synthesized by AllBio Science Incorporated (Taiwan). The purity of LP17 and control peptide are 96.21% and formula is C_89_H_137_N_23_O_25_S_1_. The peptides were synthesized by Protein Technologies Symphony^®^ Multiplex Peptide Synthesizer.

### Cell Culture

The mouse microglial cell line BV-2 was generated from primary mouse microglia transfected with a v-raf/v-myc oncogene and was maintained at 37^∘^C in Dulbecco’s modified Eagle’s medium/F12 medium (Life Technologies, Grand Island, NY, United States) with 10% heat-inactivated FBS (HyClone, Logan, UT, United States), 50 U ml^–1^ penicillin, and 50 mg/ml streptomycin (Sigma Chemical, St. Louis, MO, United States) under a humidified atmosphere of 5% CO_2_ and 95% air. Cell numbers seeded 1 × 10^6^ cells/dish in 10 cm dish for Western blot analysis and quantitative PCR (qPCR) analysis.

### Animals

The AB strain of wild-type zebrafish was used for this study. Embryos were collected after natural spawning, staged according to standard criteria, and raised synchronously at 28.5^∘^C in Hank’s buffer (13.7 mM NaCl, 540 μM KCl, 25 μM Na_2_HPO_4_, 44 μM KH_2_PO_4_, 130 μM CaCl_2_, 100 μM MgSO_4_, and 420 μM NaHCO_3_; pH 7.4). No additional maintenance was required because the embryos received nourishment from the attached yolk sac. Male Wistar rats (BioLASCO Co. Ltd., Taipei, Taiwan) at 8 weeks old, weighing 260–285 g, were used in the experiments. The rats were randomly divided into four groups: control, 6-OHDA treatment, 6-OHDA plus LP17, and LP17 alone group (five animals in each group). All studies were reported in accordance with the ARRIVE guidelines for experiments involving animals (Kilkenny et al., 2012). The rats were maintained in Plexiglas cages in a temperature-controlled (22^∘^C) room, under a 12-h light/dark cycle, and given free access to food and water.

### Ethical Approval

All animal care and experimental use of animals conformed to the Guiding Principles in the Care and Use of Animals of the American Physiology Society and was approved by the National Sun Yat-sen University Animal Care and Use Committee. All studies involving animals are reported in accordance with the ARRIVE guidelines for reporting experiments involving animals. Every effort was made to minimize both the number of animals used and their suffering. Animal use procedures were conducted in strict accordance with the NIH Guide for the Care and Use of Laboratory Animals (8th edition, 2011). Each rat was used only once during the study.

### Total RNA Extraction, Reverse Transcription, and Quantitative Real-Time PCR

Zebrafish embryos at 9 hpf were treated for 87 h with different concentrations of LP17. According to the manufacturer, total RNA was extracted from 20 zebrafish larvae of each treatment group using the TRIzol^®^ Reagent (Invitrogen^TM^, United States). RNA was reverse transcribed to a single-stranded cDNA using the iScript cDNA synthesis kit (Bio-Rad, Hercules, CA, United States). RT-PCR was performed using the gene expression assay primers for BV2 cell and zebrafish, as follows: (1) for *TREM-1*: forward: 5′-GAGCTTGAAGGATGAGGAAG-3′ and reverse: 5′-GCTCCTCCTGTGAAATAGAC-3′, (2) for *iNOS*: forward: 5′-GGAGATGCAAGGTCAGCTTC-3′ and reverse: 5′-GGCAAAGCTCAGTGACTTCC-3′, for BV2 cell as follows: (3) for *LC3-A*: forward: 5′-CGTCCTGGACAAGACCAAG-3′ and reverse: 5′-CTCGTCTTTCTCCTGCTCGT-3′, (4) for *LC3-B*: forward: 5′-AGCAGCATCCAACCAAAATC-3′ and reverse: 5′-CTGTGTCCGTTCACCAACAG-3′. We then performed real-time PCR using the iQ^TM^ SYBR^®^ Green (Bio-Rad, Hercules, CA, United States) supermix for zebrafish and cell in the Bio-Rad real-time PCR system (all materials were from Applied Biosystems). The condition of real-time PCR was listed as Table 1. The expression level of each gene was presented as relative fold change (log2 ratio), which was calculated using the comparative Ct method with GAPDH as the internal reference.

**TABLE 1:** Condition of real-time PCR.

**PCR condition**			
**Stage**	**Temperature (^∘^C)**		**Time (min:s)**
Initial denaturation	95		3:00
Denaturation	95	↓× 39 cycle	0:10
Annealing	55		0:30
Initial extension	95		0:10
Final extension	65		0:05

### Transfection of TREM-1 siRNA

The cells were aliquoted to 6-cm dishes for further experimentation. The BV2 cells were transiently transfected with specific TREM-1 siRNA (sc-43000, Santa Cruz Biotechnology, Dallas, TX, United States). The transient transfections with siRNAs were performed with siRNA Transfection Reagent (sc-29528, Santa Cruz Biotechnology, Dallas, TX, United States). Seven hours after transfection, the cultures were washed once with phosphate-buffered saline (PBS) and incubated with DMEM/F12 overnight.

### Preparation of Nuclear Extracts and Western Blotting

The extraction and isolation of nuclear fractions were performed with the ProteoJET^TM^ Cytoplasmic and Nuclear Protein Extraction Kit (Fermentas, Canada), according to the manufacturer’s instructions. The procedure was based on cell lysis with mild detergents. The prepared cell extracts were compatible with that obtained by Western blotting. An equal volume of sample buffer (2% SDS, 10% glycerol, 0.1% bromophenol blue, 2% 2-mercaptoethanol, and 50 mM Tris–HCl; pH 7.2) was added to the sample that was further loaded onto a 10% SDS–polyacrylamide gel and electrophoresed at 150 V for 60 min. After electrophoresis, the proteins were transferred onto a polyvinylidenedifluoride membrane (Immobilon-P; pore size, 0.45 μm; Millipore, Bedford, MA, United States) at 125 mA and maintained overnight at 4^∘^C in a transfer buffer (50 mM Tris–HCl, 380 mM glycine, 1% SDS, and 20% methanol). The membrane was then blocked for 50 min at room temperature (RT) with 5% non-fat dry milk and 0.1% Tween 20 in 20 mM Tris–HCl, 137 mM NaCl (TTBS, pH 7.4), and incubated with the primary antibodies for 16 h at 4^∘^C. The membrane was washed three times in TTBS for 10 min, blocked with 5% non-fat dry milk/TTBS, and incubated with the secondary antibody for 1 h at RT.

Immunoblotting was then performed using appropriate antibodies as followed: β-actin (loading control; dilution 1:1000) [Sigma, St. Louis, MO, United States; Catalog No. A5441; monoclonal antibody (mAb)]; COX-2 (dilution 1:1000) (Cayman Chemical, Ann Arbor, MI, United States; Catalog No. 160106; polyclonal antibody); histone deacetylases-6 (HDAC-6 dilution 1:1000) (GeneTex, Inc., United States; Catalog No. GTX84377; polyclonal antibody); iNOS (dilution 1:1000) (BD Pharmingen, San Diego, CA, United States; Catalog No. 6103322; polyclonal antibody); inhibitor of kappa B (IκB, dilution 1:1000) (Abcam, United States; Catalog No. ab32518; mAb); LC-3 (A/B) (microtubule-associated protein 1A/1B-light chain 3, dilution 1:1000) (Abcam, United States; Catalog No. ab58610; mAb); p-ERK (extracellular signal-related kinases; dilution 1:1500) (Cell Signaling Technology, United States; Catalog No. 9190; polyclonal antibody); p-Akt (dilution 1:1000) (Cell Signaling Technology, United States; Catalog No. 9271; polyclonal antibody); mammalian target of rapamycin (p-mTOR, dilution 1:1000) (Abcam, United States; Catalog No. ab1093; mAb); tyrosine hydroxylase (TH; dilution 1:1000) (Millipore, Bedford, MA, United States; Catalog No. MAB318; mAb); TREM-1 (dilution 1:1000) (Abcam, United States; Catalog No. ab104413; mAb); and tumor necrosis factor-alpha (TNF-α) (Abcam, United States; Catalog No. ab1793; mAb). After the incubation of primary antibody, membranes treated by horse radish peroxidase-conjugated secondary antibody. The immune reactive bands were visualized by enhanced chemiluminescence (ECL kit; Millipore, Bedford, MA, United States). The images were visualized using the UVP BioChemi Imaging System and relative densitometric quantification was performed using LabWorks 4.0 software (UVP, Upland, CA, United States). Monoclonal antibodies against β-actin were used as the internal control for protein loading and data were expressed as the ratio of the protein of interest to β-actin. The relative variations in bands between the various treatment samples and the control group were calculated using the same image.

### Locomotor Behavioral Test

Zebrafish larvae at 2 days post fertilization (dpf) were treated with 6-OHDA for 4 days in a 24-well plate with and without the tested drugs. At 5 dpf, the fishes were transferred onto 10-cm dishes (16 fishes/dish) and swimming behavior was monitored by an animal behavior system with an automated video tracking (Singa Technology Co., Taipei, Taiwan; Catalog No. TM-01) (Shen et al., 2010). Thereafter, the fishes were transferred into a quartz cuvette, which was 4.5 cm high, 1 cm wide, and 1 cm long and was housed in a distinctive plastic box measuring 16 cm in length and 4.8 cm in width. The cuvettes were placed at a distance of 7.5 cm in front of a camera (Weichu Technology Co. Ltd., Sanchong, Taiwan; Catalog No. IC-200). All instruments were adhered to a plastic plate that was 38 cm long and 19 cm wide. During a test, four cuvettes were placed in a parallel arrangement. Each zebrafish was gently placed into the tank and tracked from the side of the tank for determination of swim height. Each animal was given a 2-min adaptation period before recording the swimming pattern of each fish for 5 min. The total distance moved was defined as the length in centimeters that the fish moved during a 5-min session.

### 6-OHDA Lesions and Drug Injection to Rats

The animals (five per group) were anesthetized by inhalational anesthesia of isoflurane solution before they were secured in a Kopf stereotaxic instrument with the tooth bar set at 15 mm above the interaural line. Lesions were made by unilateral injection of 5 mg of 6-OHDA hydrochloride (10 mg in 2 ml 0.1% ascorbic acid in 0.9% saline) into the right middle forebrain bundle (MFB) at the following coordinates: AP, -1.2; ML, -2.4; DV, -8.5 mm from the bregma. The sham operated animals received only a vehicle (0.1% ascorbic acid in 0.9% saline) at the same coordinates. 6-OHDA was administered at a rate of 0.5 ml/min using a 27-G Hamilton syringe connected to an infusion minipump. The syringe was left in place for 5 min before slowly retracting it to allow toxin fusion and prevent reflux. Injection of 40 mg/kg of LP17 was given immediately before the injection of 6-OHDA at the same coordinates.

### Rotation Behavioral Testing

Two weeks after right intranigral stereotaxic injection of 6-OHDA, the animals were subjected to rotational behavior testing (Ungerstedt, 1971; Pycock, 1980; Carman et al., 1991). The rats were injected subcutaneously with D-amphetamine at 5 mg/kg and placed in a cylindrical cage that measured 240 mm in diameter and 300 mm in height. The number of both ipsilateral and contralateral rotations over a 30-min period was recorded and counted using a digital camera.

### Immunohistochemistry for Brain Tissue

Rat brain tissue was harvested, post-fixed in the same fixative for 2 h, and transferred into a 30% sucrose solution overnight at 4^∘^C. Further, 10-μm-thick sections were prepared, air-dried on microscope slides for 1 h at RT, and pre-incubated for 1 h with 4% normal goat serum diluted in 0.01% Triton X-100 and PBS. After washing three times in ice-cold PBS, the sections were incubated overnight at 4^∘^C with anti-TH (a catecholaminergic neuron cell marker; dilution, 1:1000; Millipore, Bedford, MA, United States) in 0.01% Triton X-100 and 2% normal goat serum in PBS. The sections were then made to react with rhodamine-labeled goat anti-rabbit antibody (red fluorescence; Jackson Immuno Research Laboratories Inc., United States) for 1 h at RT. Sampling location was determined for SN. We counted DAB-positive immunolabeled cells (brown) in the captured pictures in each group. Cells were counted only if they came into focus when racking the focus down through the sampling brick. Then we gained the TH-positive rate as following calculation: the sample group/control group%.

### Fluorescent Immunohistochemistry

Cultures and tissues were fixed with 4% paraformaldehyde for 10 min at RT. Cultures were incubated in blocking buffer for 1 h and then in a 1 μg/ml dilution of anti-OX-42 (CD11b, microglia marker, 1:200, Cat. CBL1512; EMD Millipore, Temecula, CA, United States; monoclonal mouse antibody) and anti-glial fibrillary acidic protein (anti-GFAP; astrocyte marker, 1:200, Cat. MAB3402; EMD Millipore, Temecula, CA, United States; monoclonal mouse antibody) antibody for 15 h. The cultures were replaced with secondary antibody (Alexa 488 anti-mouse IgG, 1 μg/ml; Molecular Probes, Eugene, OR, United States) for 2 h at RT. Cultures were rinsed with Tris Buffered Saline (TBST) as described earlier, mounted on glass slides (Fisher Scientific, Waltham, MA, United States) with SlowFade Light (Molecular Probes, Eugene, OR, United States), and visualized using a Leica Optical Confocal Laser Scanning Microscopy System (Leica, DM6000B) with the SPOT program (Diagnostic, RT slider SPOT, Hamilton, CA, United States). For immunostaining analysis, stained brain sections were examined using a Leica DM-6000 CS fluorescence microscope (Leica Instruments Inc., Wetzlar, Germany). The pixel values of the immunoreactive-positive area were counted using Image J software (National Institutes of Health, Bethesda, MD, United States) using the sections of SN, and expressed as a percentage change compared to the 6-OHDA group, which were considered to be 100%.

### Assay for Soluble TREM-1 (sTREM-1) Concentration

We determined soluble TREM-1 (sTREM-1) concentration which was referred by published methods (Gibot et al., 2005b; Kamei et al., 2010). Blood was taken by cardiac puncture and immediately placed into iced heparinized tubes containing an excess of DPP4 inhibitor. These were directly centrifuged (1500 × *g* at 4^∘^C). The plasma was then removed and immediately frozen at −80^∘^C. Samples were later thawed on wet ice for the further analysis. sTREM-1 concentrations were determined using an enzyme-linked immunosorbent assay kit (R&D Systems, Inc., Minneapolis, MN, United States) designed for the quantification of mouse/rat TREM-1 concentrations in serum. Briefly, samples were incubated for 2 h at RT in wells coated with a mAb specific for TREM-1. After the wells were washed four times, a peroxidase-conjugated anti-TREM-1 polyclonal antibody was added to the wells and incubated for 2 h at RT. After four washings, luminogen was added and incubated for 30 min at RT. A stop solution was added to terminate the enzyme reaction. The optical density (OD) was measured at 450 nm using a microplate reader. The concentration of rat TREM-1 was calibrated from a dose–response curve based on reference standards. The assay was performed in duplicate. In this assay, the intra-assay coefficient of validation (CV) and inter-assay CV were <5% and approximately 10%, respectively.

### Statistical Analysis

All experiments were repeated at least three times. All data were represented as mean ± SEM. For immunoreactivity data, the intensity of each test band was expressed as the relative OD, which was calculated from the average OD values obtained from all controls. Whenever applicable, data were analyzed using two-way ANOVA followed by Dunnett’s test when found to be significant. Repeated measures were used to analyze the group differences the factors used were LPS or 6-OHDA and LP17.

## Results

### The Effect of LP17 on TREM-1 Receptor Expression in Murine Microglia

**FIGURE 1:**
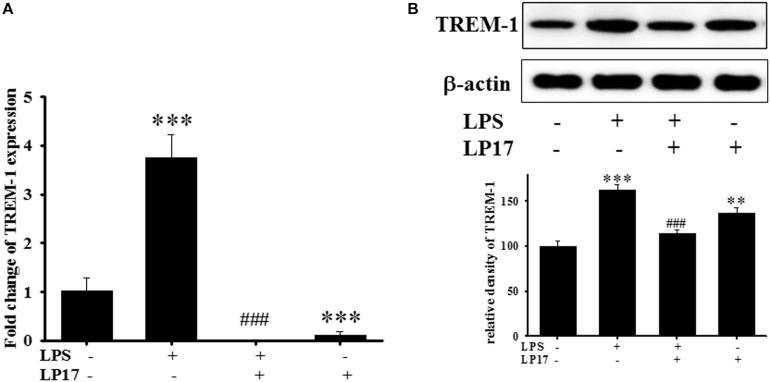
The treatment of LP17 for 16 h attenuated LPS-induced upregulation of TREM-1 mRNA and protein expressions in BV2 murine microglial cell line (*n* = 4/group). As shown on **(A)** quantitative PCR, **(B)** at the protein level by Western blotting. Co-treatment with 10 μg/ml LP17 significantly attenuated the LPS-induced upregulation of TREM-1 in mRNA and protein level. The bars represent the mean ± SEM. The data were analyzed using two-way ANOVA. ^∗∗^*P* < 0.05; ^∗∗∗^*P* < 0.001 versus the control group; ^###^*P* < 0.001 versus the LPS group.

We examined the effect of LP17 on LPS-induced upregulation of TREM-1 expression in BV-2 murine microglia cells. On qPCR analysis, co-treatment with 10 mg/ml LP17 for 16 h significantly attenuated LPS-induced upregulation of TREM-1 mRNA expression (Figure 1A). Likewise, on Western blotting, LP17 significantly attenuated LPS-induced upregulation of TREM-1 expression (Figure 1B). Uncropped Western blots of TREM-1 and β-actin were shown in Supplementary Figure S1.

### LP17 Inhibits LPS-Induced Upregulation of iNOS, COX-2, IκB, and NF-κB Protein Expression in BV-2 Murine Microglial Cells

**FIGURE 2:**
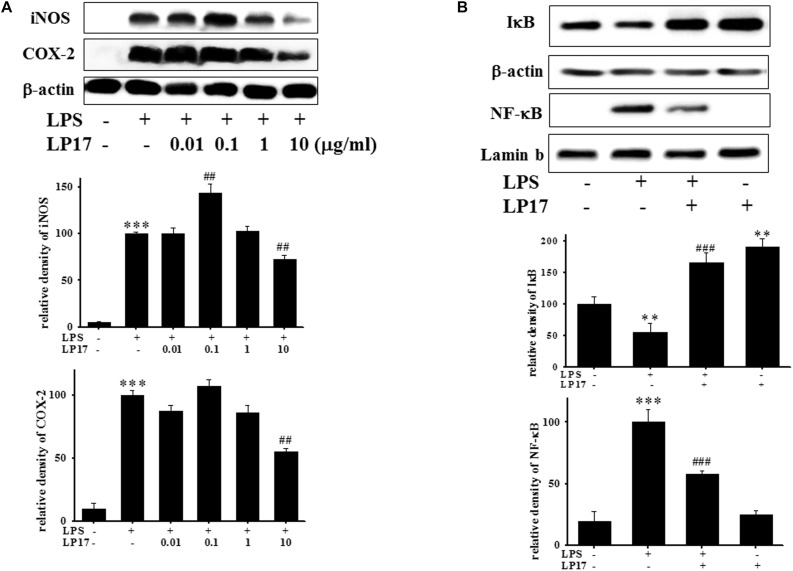
The treatment of LP17 inhibited LPS-induced upregulation of expressions of proinflammatory cytokines iNOS, COX-2, IκB, and NF-κB protein in LPS-stimulated BV-2 murine microglial cell line (*n* = 4/group). **(A)** Western blotting for iNOS, COX-2 expression after treatment of BV-2 microglial cells with 0.01, 0.1, 1, and 10 μg/ml LP17 plus 100 ng/ml LPS for 16 h, showed that 10 μg/ml LP17 significantly inhibited LPS-induced increase in iNOS and COX-2 protein expressions. **(B)** Western blotting for IκB, and nuclear NF-κB after treatment of BV-2 microglial cells with 10 μg/ml LP17 plus 100 ng/ml LPS for 6 h, showed that LP17 significantly reversed the LPS-induced downregulation of IκB protein expression and significantly attenuated the LPS-induced upregulation of nuclear NF-κB protein expression. The bars represent the mean ± SEM. The data were analyzed using two-way ANOVA. ^∗∗^*P* < 0.05; ^∗∗∗^*P* < 0.001 versus the control group; ^##^*P* < 0.05, ^###^*P* < 0.001 versus the LPS group.

We further evaluated the effects of LP17 on iNOS and COX-2 expression in LPS-stimulated BV-2 microglia. Co-treatment with 10 μg/ml LP17 for 16 h significantly attenuated LPS-induced upregulation of iNOS and COX-2 expressions (Figure 2A). LP17 treatment for 6 h markedly reversed LPS-induced downregulation of IκB protein levels and significantly attenuated LPS-induced upregulation of nuclear NF-κB expression (Figure 2B). Uncropped Western blots of iNOS, COX-2, IκB, NF-κB, lamin-b, and β-actin were shown in Supplementary Figures S2, S3.

### LP17 Enhanced LPS-Induced Upregulation in the Autophagy Pathway in Microglia

**FIGURE 3:**
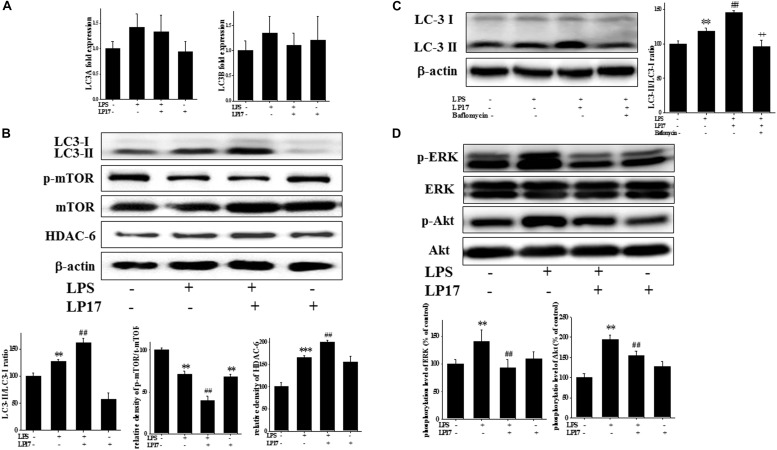
Treatment with LP17 enhanced LC3-II/LC3-I, HDAC6, and p-mTOR expression and inhibited p-ERK and p-Akt protein expression in an LPS-stimulated murine BV-2 microglial cell line (*n* = 4/group). **(A)** Quantitative PCR for LC3A and LC3B in the control, LPS, LPS plus LP17, and LP17-only groups after BV-2 microglial cells were treated with 10 μg/ml LP17 plus 100 ng/ml LPS for 6 h. Neither LP17 nor LPS affected LC3A and LC3B mRNA expression. **(B)** Western blot analysis for LC3, HDAC6, and p-mTOR expression in the control, LPS, LPS plus LP17, and LP17-only groups after the treatment of BV-2 microglial cells with 10 μg/ml LP17 plus 100 ng/ml LPS for 16, 16, and 2 h, respectively. LP17 significantly enhanced the LPS-induced increase in the LC-3-II/LC-3-I ratio, attenuated the LPS-induced down-regulation of p-mTOR, and enhanced the LPS-induced upregulation of HDAC6 protein expression. **(C)** Western blot analysis for LC3-II/LC3-I ratio in the control, LPS, LPS plus LP17, and LPS plus LP17 + pretreatment of bafilomycin groups after BV-2 microglial cells were treated with 10 μg/ml LP17, 100 ng/ml LPS plus 5 nM bafilomycin for 16 h. Pretreatment with bafilomycin for 1 h significantly inhibited the LP17-induced increase of the LC3-II/LC3-I ratio. **(D)** Western blot analysis for p-Akt and p-ERK after the treatment of BV-2 microglial cells with 10 μg/ml LP17 plus 100 ng/ml LPS for 16 h. The results showed that LP17 significantly inhibited the LPS-induced upregulation of the p-Akt and p-ERK proteins. The bars represent means ± SEM. The data were analyzed using two-way ANOVA. ^∗∗^*P* < 0.05; ^∗∗∗^*P* < 0.001 versus the control group; ^##^*P* < 0.05 versus the LPS group; ^++^*P* < 0.05 versus the LPS plus LP17 group.

We investigated the role of the autophagy pathway in BV-2 cells. Co-treatment with 10 μg/ml LP17 for 6 h had no effect on LC3A and LC3B mRNA expression (Figure 3A). Co-treatment with LP17 for 16 h significantly enhanced the LPS-induced increase in the LC3-II/LC3-I ratio, whereas treatment with LP17 for 16 h significantly reversed the LPS-induced downregulation of HDAC-6 expression. Moreover, 1 h of LP17 treatment clearly attenuated the LPS-induced downregulation of p-mTOR expression (Figure 3B). Pretreatment with 5 nM bafilomycin for 1 h significantly inhibited the LP17-induced enhancement of the LC3-II/LC3-I ratio (Figure 3C). The combined treatment of BV-2 cells with LP17 and LPS for 1 h resulted in a marked inhibition of ERK and Akt activation by LP17 (Figure 3D). Uncropped Western blots of LC3, p-mTOR, HDAC-6, mTOR, p-ERK, ERK, p-Akt, Akt, and β-actin are shown in Supplementary Figures S4, S5.

### Effect of TREM-1 Knockdown on TREM-1, iNOS, COX-2, IκB, HDAC6, and LC3 Expression in BV2 Cells

**FIGURE 4:**
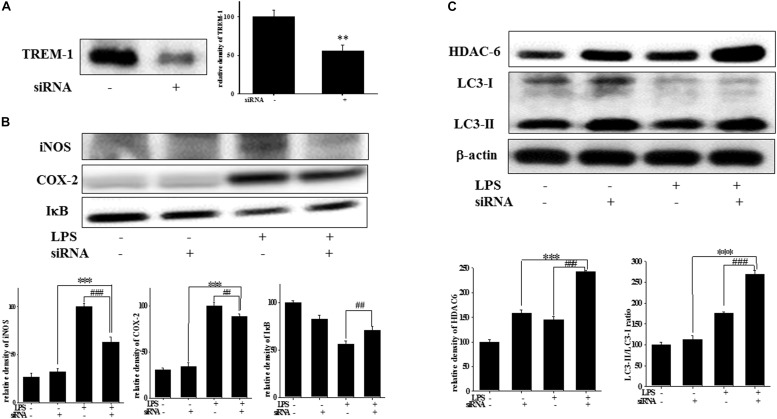
TREM-1 knockdown in BV2 cells decreased TREM-1 expression levels and the LPS-induced upregulation of iNOS, COX-2, and IκB expression and increased HDAC6 expression and the LC3-II/LC3-I ratio. **(A)** Western blot analysis of TREM-1 expression in the control siRNA and TREM-1 siRNA groups for 7 h (*n* = 4/group). The results demonstrate that the transfection of TREM-1 siRNA significantly attenuated the expression of TREM-1. **(B)** Western blot analysis for iNOS, COX-2, and IκB expression in TREM-1 knockdown BV2 cells in the control (control siRNA), LPS-treated (control siRNA), control (TREM-1 siRNA), and LPS-treated (TREM-1 siRNA) groups after treatment with 100 ng/ml LPS for 16 h (*n* = 4/group). The knockdown of TREM-1 in BV2 cells attenuated the LPS-induced upregulation of iNOS and COX-2 and reversed the LPS-induced downregulation of IκB expression. **(C)** Western blot analysis for HDAC-6 expression and LC3-II/LC3-I ratio in TREM-1 knockdown BV2 cells in the control (control siRNA), LPS-treated (control siRNA), control (TREM-1 siRNA), and LPS-treated (TREM-1 siRNA) groups after treatment with 100 ng/ml LPS for 16 h (*n* = 4/group). Knockdown of TREM-1 also enhanced the LPS-induced upregulation of HDAC6 and increased the LC3-II/LC3-I ratio. “–” represents control siRNA and “+” represents TREM-1 siRNA. The bars represent means ± SEM. ^∗∗∗^*P* < 0.001 versus the control group (TREM-1 siRNA); ^##^*P* < 0.05 versus the LPS group (control siRNA); ^###^*P* < 0.001 versus the LPS group (control siRNA).

**FIGURE 5:**
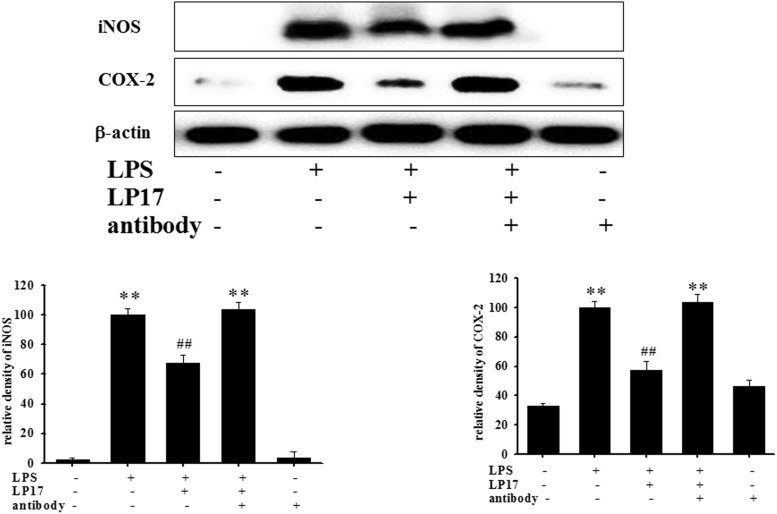
The treatment of TREM-1 agonist antibody reduced the anti-inflammatory effects of LP17 on iNOS and COX-2 expressions in LPS-stimulated murine BV-2 microglial cell line (*n* = 4/group). Western blotting for iNOS and COX-2 expression of control, LPS, LPS plus LP17, LPS plus LP17 + agonist antibody, and agonist antibody group after the BV-2 microglial cells were treated with 10 μg/ml LP17 plus 100 ng/ml LPS for 6 h and 30 min, followed by the addition of 5 μg/ml TREM-1 agonist antibody to the solution for 16 h. The agonist antibody significantly inhibited the anti-inflammatory effect of LP17 on LPS-induced upregulation of iNOS and COX-2 expressions. The bars represent the mean ± SEM. The data were analyzed using two-way ANOVA.^∗∗^*P* < 0.05 versus the control group; ^##^*P* < 0.05 versus the LPS group.

At 7 h post-siRNA transfection, TREM-1 expression levels were reduced by 55.9% (Figure 4A). Compared with the expression in normal cells, iNOS and COX-2 expression was significantly decreased and IκB expression was significantly increased in TREM-1 knockdown cells in the LPS group (Figure 4B). Moreover, in BV2 cells, TREM-1 knockdown increased HDAC6 expression and the LC3-II/LC3-I ratio (Figure 4C), while partially inhibiting the inflammation process and increasing autophagy. Uncropped Western blots of TREM-1, iNOS, COX-2, IκB, LC3, HDAC6, and β-actin are shown in Supplementary Figures S6, S7. Co-treating BV-2 cells with TREM-1 agonist antibody (5 μg/ml), LP17 (10 μg/ml), and LPS for 16 h eliminated the effect of LP17 on the reduction of iNOS and COX-2 expression in LPS-treated BV-2 cells (Figure 5). Uncropped Western blots of iNOS, COX-2, and β-actin are shown in Supplementary Figure S8.

### The Effects of LP17 on Locomotor Activity, mRNA, and Protein Expressions in 6-OHDA-Treated Zebrafish

**FIGURE 6:**
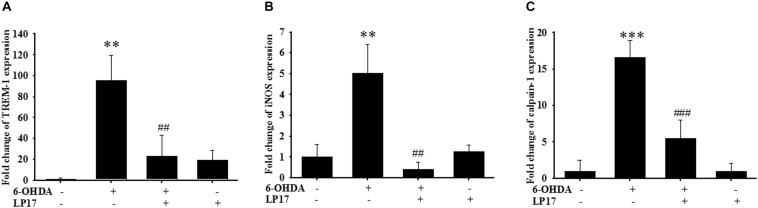
LP17 attenuated the 6-OHDA-induced upregulation of TREM-1 and iNOS expressions in a zebrafish PD model (*n* = 3/group). **(A–C)** The quantitative PCR of TREM-1, iNOS and calpain-1 expression of control, 6-OHDA, 6-OHDA plus LP17, and LP17 group after the zebrafish was treated with 0.1 μg/ml of LP17 (from 9 hpf to 4 dpf) and 250 μM 6-OHDA (from 2 to 4 dpf) at 4 dpf. LP17 attenuated the 6-OHDA-induced increase in TREM-1, iNOS, and calpain-1 mRNA expressions. Each sample contained 20 zebrafish heads. The bars represent the mean ± SEM. The data were analyzed using two-way ANOVA. ^∗∗^*P* < 0.05 versus the control group; ^##^*P* < 0.05 versus the 6-OHDA group.

**FIGURE 7:**
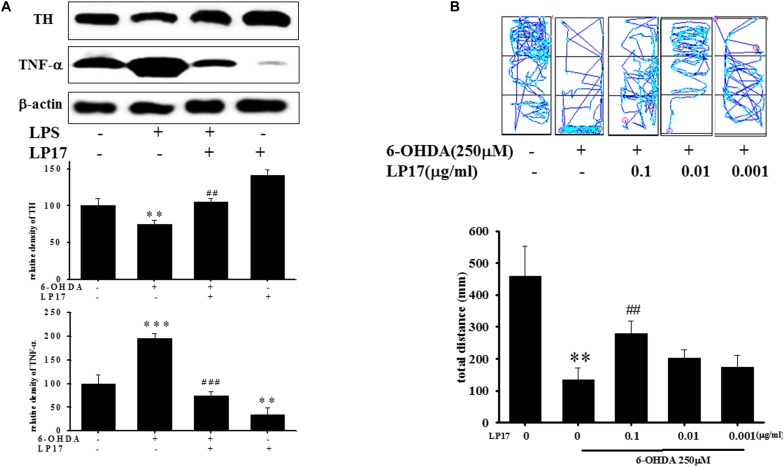
Treatment of LP17 reversed 6–OHDA–induced downregulation of TH expression, upregulation of TNF-α expression, and locomotor deficit. **(A)** Western blot analyses for TH and TNF-α expression at 5 dpf of control, 6-OHDA, 6-OHDA plus LP17, and LP17 group after the zebrafish were treated with 0.001, 0.01, and 0.1 μg/ml LP17 (from 9 hpf to 4 dpf) and with 250 μM 6–OHDA (from 2 to 4 dpf) (*n* = 3/group). It showed that LP17 significantly reversed the 6-OHDA-induced downregulation of TH and attenuated the 6–OHDA–induced upregulation of TNF-α expression. Each sample contained 20 zebrafish heads. **(B)** Determination of the typical swimming pattern and total swimming distance of zebrafish larvae at 5 dpf in the control, 6–OHDA, 6–OHDA plus LP17, and LP17 groups (*n* = 16/group) shows that 0.1 μg/ml of LP17 significantly reversed the 6–OHDA–induced deficiency of locomotor activity in 5 dpf. The bars represent the mean ± SEM. The data were analyzed using two-way ANOVA. ^∗∗^*P* < 0.05, ^∗∗∗^*P* < 0.001 versus the control group; ^##^*P* < 0.05; ^###^*P* < 0.001 versus the 6-OHDA group.

We examined the effects of LP17 on zebrafish in a PD model. The zebrafish were treated with 0.1 μg/ml LP17 (from 9 hpf to 3 dpf) and 250 μM 6-OHDA (from 2 to 3 dpf). We used quantitative real-time PCR to measure TREM-1-related gene expression in 6-OHDA-treated zebrafish. Pretreatment with 0.1 μg/ml LP17 attenuated the 6-OHDA-induced upregulation of TREM-1 (Figure 6A), iNOS (Figure 6B), and calpain-1 (Figure 6C) mRNA expressions. Moreover, pretreatment with LP17 reversed the 6-OHDA-induced downregulation of TH expression and markedly attenuated the 6-OHDA-induced upregulation of TNF-α protein expression (Figure 7A). The zebrafish were treated with 0.1, 0.01, and 0.001 μg/ml of LP17 (from 9 hpf to 4 dpf) and with 250 mM of 6-OHDA (from 2 to 4 dpf). Our data demonstrated that pretreatment with LP17 significantly rescued the locomotor deficits induced by 6-OHDA in zebrafish larvae in 5 dpf (Figure 7B). Uncropped Western blots of TH, TNF-α, and β-actin are shown in Supplementary Figure S9.

### LP17 Treatment Partially Protects Dopaminergic Neurons Against 6-OHDA Lesion

**FIGURE 8:**
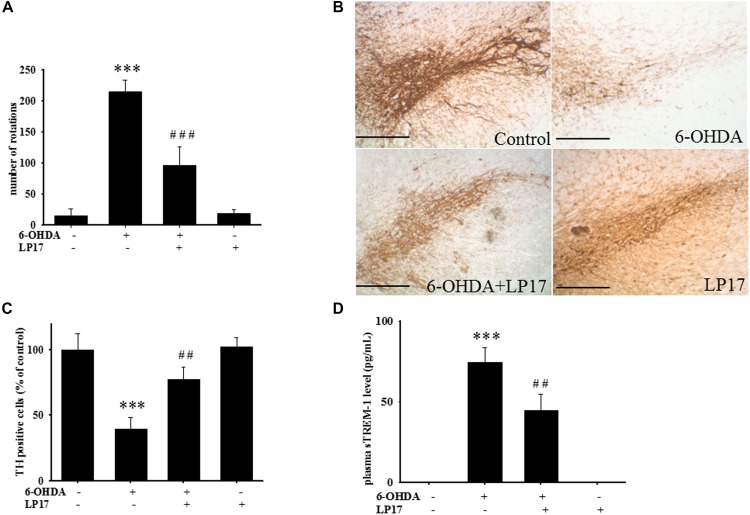
Treatment of LP17 rescued 6-OHDA-induced upregulation of the number of amphetamine-induced rotation behavior, downregulation of TH expression, and upregulation of plasma sTREM-1 in rat model (*n* = 5/group). **(A)** Analyses of the number of rotations at 14 days after development of lesions in the 6-OHDA group and the 6-OHDA plus LP17 group after the rats were treated with either the control peptide or 40 μg/kg of LP17 (1 μg/μl for a 10-μl solution) plus 5 μg 6-OHDA. It showed that LP17 significantly reversed the 6-OHDA-induced increase in the number of rotation behaviors. **(B,C)** Whole-mount immunohistochemistry and quantitative result of TH-positive cells for TH expression at 14 days after development of lesions in the control, 6-OHDA, 6-OHDA plus LP17, and LP17 alone groups. LP17 significantly reversed the 6-OHDA-induced downregulation of TH (scale bar = 100 μm). **(D)** Based on the plasma level of sTREM-1 at 14 days after development of lesions in the control, 6-OHDA, 6-OHDA plus LP17, and LP17 alone groups, LP17 significantly attenuated the 6-OHDA-induced upregulation of sTREM-1 in plasma. The bars represent the mean ± SEM. The data were analyzed using two-way ANOVA. ^∗∗^*P* < 0.05; ^∗∗∗^*P* < 0.001 versus the control group; ^##^*P* < 0.05; ^###^*P* < 0.001 versus the 6-OHDA group.

Two weeks after co-treatment of 20 μg/kg 6-OHDA with 40 μg/kg LP17, the ipsilateral rotations induced by 5 mg/kg methamphetamine were tested and showed that pharmacologic treatment affected the number of rotations for 30 min. Intracerebroventricular (ICV) injection of LP17 significantly attenuated the 6-OHDA-induced upregulation of the number of rotations (Figure 8A).

**FIGURE 9:**
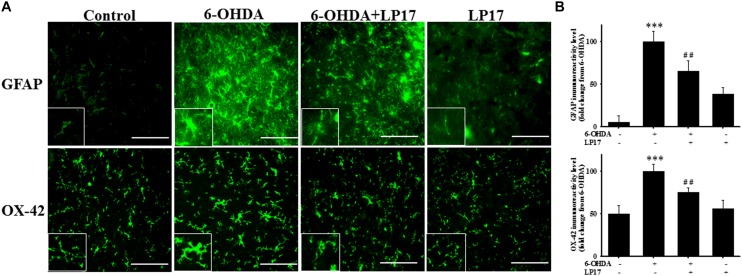
Treatment with LP17 attenuated the 6-OHDA-induced upregulation of OX-42 and GFAP expression in a rat model of PD (*n* = 5/group). **(A)** Analyses of GFAP and OX-42 immunoreactivity at 14 days after the development of lesions in the 6-OHDA group and the 6-OHDA plus LP17 group after treatment with either the control peptide or 40 μg/kg LP17 (1 μg/μl for a 10-μl solution) plus 5 μg 6-OHDA. The results showed that LP17 significantly attenuated 6-OHDA-induced increases in GFAP and OX-42 expression. **(B)** Quantitative results of immunoreactivity analyses for GFAP and OX-42 expression at 14 days after the development of lesions in the control, 6-OHDA, 6-OHDA plus LP17, and LP17-only groups. LP17 significantly decreased 6-OHDA-induced GFAP and OX-42 upregulation (scale bar = 25 μm). The bars represent means ± SEM. The data were analyzed using two-way ANOVA. ^∗∗∗^*P* < 0.001 versus the control group; ^##^*P* < 0.05 versus the 6-OHDA group.

Stereologic counting of TH-immunopositive neurons in the SN revealed a significant effect of LP17 treatment (i.e., 60.9% loss caused by 6-OHDA). In particular, LP17 treatment significantly reversed the 6-OHDA-induced reduction of the number of TH-immunopositive neurons (Figures 8B,C). Co-treatment with LP17 significantly attenuated 6-OHDA-induced upregulation of sTREM-1 (Figure 8D). Furthermore, fluorescent immunohistochemical analyses of GFAP and OX-42 were performed after 2 weeks of 6-OHDA administration. LP17 significantly attenuated the 6-OHDA-induced upregulation of GFAP and OX-42 immunoreactivity (Figure 9A). The immunoreactivity of GFAP in the control, 6-OHDA, 6-OHDA + LP17, and LP17 treatment groups was 5.3 ± 7.5, 100.0 ± 12.2, 65.3 ± 12.0, and 38.2 ± 8.2, respectively (Figure 9B), and the immunoreactivity of OX-42 in the control, 6-OHDA, 6-OHDA + LP17, and LP17 treatment groups was 50.3 ± 9.5, 100.0 ± 8.3, 75.3 ± 5.4, and 56.3 ± 9.4, respectively (Figure 9B).

## Discussion

### Summary of Findings

A large body of research on the treatment of PD has focused on the inhibition of specific downstream inflammatory mediators such as COX-2 and NF-κB (Chen et al., 2000; Steinbach et al., 2000; Cho et al., 2004). However, only a few studies have investigated the effects of modulating upstream inflammation amplifiers, such as the TREM family, in NDs (Bouchon et al., 2001a; Kemna et al., 2005). Therefore, understanding the contribution of proximal pathways that amplify the inflammatory process could aid in the development of targeted therapies that limit injury and inflammation in this devastating CNS disease.

To the best of our knowledge, our study is the first to identify the therapeutic effects of TREM-1 receptor blockage in PD. We confirmed that the TREM-1 receptor is expressed on BV2 microglial cells (Figure 1). We also found that LP17, a TREM-1 receptor antagonist, attenuates TREM-1 expression in mRNA and protein level. A previous study indicated that TREM-1 and its ligand have a synergistic effect with LPS and an amplified synthesis of the proinflammatory cytokines TNF-α and GM-CSF (Bleharski et al., 2003). We attenuated the inflammation effect with LP17 induced by LPS which could also inhibit the expression of TREM-1. LP17 attenuated the LPS-induced upregulation of proinflammatory cytokine expression (i.e., NF-κB, iNOS, and COX-2) in microglia (Figure 2). LP17 also enhances autophagy-related protein expression (Figure 3). We then examined the effect of LP17 on autophagy after treatment with bafilomycin, an inhibitor of autolysosome, and found that treatment of bafilomycin inhibits LPS- and LP17-induced up-regulation of LC3-II/LC3-I ratio (Figure 3). Besides, we found that the knockdown of TREM-1 in BV2 cells alleviates inflammation and enhances autophagy (Figure 4). We further confirmed the neuroprotective activity and anti-inflammatory effects of TREM-1 inhibition in a zebrafish PD model (Figures 6, 7). The incubation times of LP17 and 6-OHDA were referred the conditions of experience in previous studies (Feng et al., 2014, 2016). In Feng et al. (2014), we treated zebrafish with different doses of 6-OHDA and found a significant reduction in zebrafish locomotor activity using an optimal 250 μM concentration of 6-OHDA. We also found that 3 days (2 dpf to 5 dpf) of 6-OHDA treatment decreased the total swimming distance. We also tested a variety of potential compounds or clinical drugs involved in targeting factors such as Vitamin E, minocycline, and Sinemet from 9 hpf to 5 dpf (Feng et al., 2014). The appropriate concentration of LP17 was determined by the survival rate from 0.001 to 0.1 μg/ml were all 100% in 5 dpf and the locomotor activity test in Figure 7B. Moreover, antagonizing TREM-1 clearly inhibited the upregulation of 6-OHDA-induced changes in rat behavior (Figure 8). The treatment time in rat experiments was described in previous studies (Feng et al., 2016, among others). We treated concomitantly with either 20 μg/kg (concentration 0.5 μg/μl for 10 μl) 11-dehydrosinulariolide or vehicle (0.1% ascorbic acid in 0.9% saline), and 5 μg 6-OHDA (Feng et al., 2016). We also used other studies for reference (Sharifi et al., 2013; Yu et al., 2017). Finally, the overactivation of microglia and astrocytes was confirmed in a rat PD model, and LP17 was found to significantly attenuate the 6-OHDA-induced activation of these cells (Figure 9).

### The Expression of TREM-1 and TREM-2 in Microglia

Some previous studies indicated that the TREM-1 receptor was mainly expressed in monocytes, macrophages, and neutrophils (Bouchon et al., 2001a, b). However, Jiang et al. (2016) indicated that TREM-1 was expressed by circulating monocytes and microglia and is coupled with TYRO protein tyrosine kinase binding protein (TYROBP) to amplify inflammatory responses (Jiang et al., 2016). In addition, our results also revealed that the number of TREM-1-positive cells in microglia increased after treatment with LPS, regardless of the absence or presence of LP17 (Figure 1). We suggested that TREM-1 could not only express in microglia but also regulate its own expression, similar to a research in macrophage (Arts et al., 2013). Few studies investigated the role of TREM-1 in the CNS (Aldrich and Kielian, 2011), whereas most brain-related studies focused on TREM-2 (Lue et al., 2015; Ulrich et al., 2017; Yeh et al., 2017) and its mutations that could lead to a chronic ND, named Nasu–Hakola disease (Nguyen et al., 2002). A deficient TREM-2/DAP12 pathway was reported to enhance the development of NDs (Paloneva et al., 2000, 2002). However, Charles et al. (2008) showed that TREM-2 signaling triggered the phagocytic uptake of cellular debris and was associated with further downstream induction of accumulation of ROS and the pro-inflammatory transcription factor NF-κB (Charles et al., 2008). The controversial role of TREM-2 in the CNS makes it inappropriate as a therapeutic target.

On the contrary, all researches pointed out that activation of TREM-1 could enhance the downstream inflammation process. Our data also confirmed that the situation in CNS system remains unknown. The treatment with LP17 inhibits the inflammation process induced by LPS in microglia. The therapeutic efficacy in the zebrafish and rat PD models strongly supported us in further clarifying its mechanism of actions. Our study also provided evidence for the hypothesis that the inhibition of inflammatory processes by LP17 could provide neuroprotection against PD. These results revealed that TREM-1 might play an important role in neuroinflammation in PD. Afterward, we further investigated the TREM-1 downstream pathway included Akt and ERK pathway.

### TREM-1 and Its Downstream Akt and ERK Pathways Are Involved in the CNS TREM-1 Cascade

Previous studies indicated that activation of TREM-1 and TLR4 could mediate the activation of interleukin 1 receptor-associated kinase 1 (IRAK1), which could be directly activated by TREM-1 or DAP12 after recruitment by lipid raft. The activation of IRAK1 leads to phosphorylation of NF-κB via the Akt pathway (Fortin et al., 2007). Moreover, activation of the TREM-1/DAP12 cascade further activates the downstream Btk and lead to significant upregulation of calcium influx, PLCγ, and ERK (Arts et al., 2013). Our data revealed the same trend in microglia as previous reports in macrophage. Blockage of TREM-1 could significantly attenuate the LPS-induced up-regulation of p-ERK and p-Akt expression. These two proteins could further modulate downstream two pathways including autophagy and neuroinflammation.

### Relationship Between Inflammation, TREM-1, and Autophagy

A direct relationship between autophagy and inflammation was recently reported, with TREM-1 being affected by TLR4 recruitment and TLR stimuli possibly inducing autophagy. Sanjuan et al. (2007) indicated autophagy as one of the downstream responses of TLR4 signaling (Sanjuan et al., 2007) and is a conserved response to stress. Different TLR agonists, such as the TLR4 agonist LPS, have been reported to induce autophagic signaling in macrophages (Xu et al., 2007; Delgado et al., 2008). Likewise, our results showed that administration of LPS significantly enhanced the autophagy-related proteins. LPS served to induce inflammation and autophagy may play an important role in the regulation of inflammation. Besides, the anti-inflammatory functions of autophagy involve prevention of inflammasome activation and downregulation of the response once an inflammasome is activated (Saitoh et al., 2008a; Nakahira et al., 2011; Zhou et al., 2011; Deretic et al., 2015). In addition, many studies demonstrated the protective effect of enhancement on autophagy in PD (Jang et al., 2016; Xu et al., 2017; Fowler and Moussa, 2018). However, few studies investigated the direct relationship between TREM-1 and autophagy. A recent study revealed that blockade of TREM-1 significantly restored an impaired autophagy activity, reduced colitis in mice, and prevented chronic inflammation by modulating autophagy and ER stress and by preventing dysbiosis (Kökten et al., 2017). Our data also revealed the same trend in the CNS system, the result depicted that blockade of TREM-1 with LP17 could enhance autophagy process to modulate neuroinflammation. Except for the *in vitro* confirmation, we also proved it in rat and zebrafish PD model. Our results showed that the administration of 6-OHDA significantly activated microglia and astrocytes, an outcome similar to that of previous studies (Haas et al., 2016; Tentillier et al., 2016). To our excitement, we found that treatment with LP17 significantly attenuated the 6-OHDA-induced activation of microglia and astrocytes. Apart from neuroinflammation, we were also interested in the relationships with TREM-1 and autophagy.

### Relationship Between Autophagy-Related Protein and TREM-1

Autophagy is mediated by increases in ATG7 and ATG16 and further activates the downstream molecules, such as LC3-I transfer to LC3-II. This process could give rise to the formation of autophagosomes (Klionsky et al., 2011; Yang et al., 2015). Guo et al. (2013) showed that tumor-specific deletion of ATG7 could increase the expression of TREM-1. In addition, mTOR was reported to modulate TREM-1 expression. Lee et al. (2015) showed that TREM-1 expression in human macrophage was partially inhibited by rapamycin, a mTOR inhibitor, and that vitamin D induced the expressions of TREM-1 and hypoxia-inducible factor 1 via mTOR signaling. Our current results showed that treatment with LPS caused modulation of autophagy-related proteins, such as LC3-II, HDAC6, and p-mTOR. The inhibition of TREM-1 with LP17 could significantly enhance autophagy and eliminate inflammatory cytokines for maintenance of cell homeostasis in microglia. The inhibition of the inflammation process induced by autophagy was widely discussed in neurological disorders.

### The Relationship Between Autophagy and Inhibition of Neuroinflammation in PD

Autophagy has been largely investigated in neurons, where it plays a crucial role as a checkpoint for protein and organelle quality control. Thus, the specific deletion of pivotal autophagic genes such as ATG-7 (Komatsu et al., 2006) or ATG-5 (Hara et al., 2006) from the neural lineage induces the formation of inclusion bodies and neurodegeneration in the absence of pathological disorder. As such, amounts of evidence indicate that autophagy in the CNS plays a major role in the promotion of neuronal health and survival. The studies in PD also suggest that autophagy may regulate microglial inflammation (Plaza-Zabala et al., 2017; Han et al., 2019). Indeed, intraventricular MPP^+^ in rats enhances active caspase-1 and cathepsin B levels in nigral microglia (Hung et al., 2016). Interestingly, the anti-inflammatory phenolic flavonoid, baicalein, attenuates inflammation and up-regulates LC3-II levels in the SN of MPP + injected rats (Hung et al., 2016), suggesting that autophagy modulation regulates the microglial inflammatory response in PD. Another recent study has also shown that metformin, an MTORC1 inhibitor currently used to treat type 2 diabetes, may activate autophagy flux in the SN of MPTP mouse model of PD. Metformin attenuates inflammasome activation and pro-inflammatory cytokine TNF-α and IL-6 levels while increasing anti-inflammatory IL-10 levels, suggesting that autophagy may control the SN inflammatory response in an *in vivo* model of PD. However, this report did not provide evidence of the specific role of microglia on MPTP-elicited PD-like inflammatory response. As such, further studies are needed to unravel the potential involvement of autophagy in PD-related microglial neuroinflammation (Lu et al., 2016). The studies mentioned above directly showed the role of autophagy played in inhibition of neuroinflammation. Our data showed that the enhancement of autophagy induced by LP17 can inhibit the neuroinflammation directly and the inhibition of neuroinflammation showed neuroprotective efficacy in two *in vivo* model. Moreover, the we also modulate activity of TREM-1 with siRNA and agonist antibody to further confirm the role of TREM-1 in neuroinflammation process.

### TREM-1 Agonist Antibody and Knockdown in Peripheral Research

A previous study demonstrated that TREM-1 knockdown decreases levels of inflammatory cytokines such as TNF-α, IL-1β, and IL-6 in LPS-induced U937 cells (Fan et al., 2016). Our results showed the same trend in microglia. We knocked down TREM-1 expression in murine microglial cells to further confirm the role that TREM-1 plays in neuroinflammation. Similar to the result of antagonist peptide LP17, TREM-1 knockdown significantly inhibited the LPS-induced inflammation process and enhanced autophagy. Except for the elimination of TREM-1 activity, many studies use agonist antibody to enhance TREM-1 action (Amatngalim et al., 2011; Weiss et al., 2017; Yuan et al., 2017). As shown in previous studies, TREM-1 acts as an inflammation amplifier that may enhance LPS-induced inflammation processes, and TREM-1 is upregulated in the presence of LPS-treated monocytes. Notably, TREM-1 synergizes with LPS to induce the secretion of proinflammatory cytokines such as TNF-α and IL-1β but does not cause a significant change in TREM-1 mAb alone group (Bouchon et al., 2001a). Furthermore, the same trend was confirmed not only in monocytes, but also in macrophages. Murakami et al. (2006) demonstrated that TREM-1 mAb may also synergistically enhance the production of proinflammatory cytokines by macrophages but not cause significant release of pro-inflammatory cytokine in TREM-1 mAb alone (Murakami et al., 2006). Our data showed a similar trend as previous reports. The treatment of TREM-1 mAb significantly abolished anti-inflammation effect from LP17, the TREM-1 mAb alone didn’t cause significant change in both iNOS and COX-2 expression. Except for the original form of TREM-1, some research also detected sTREM-1 in human (Arguinano et al., 2017).

### Relationship Between sTREM-1 Level and Inflammatory Disease

Some studies have indicated that cells release the soluble form of TREM-1 (Bouchon et al., 2001a), and clinical studies have reported the detection of sTREM-1 in patient serum (Gibot et al., 2004c). sTREM-1 in body fluid is reportedly useful as a diagnostic marker of infectious inflammatory conditions, particularly in severe sepsis (Gibot et al., 2004a, 2005a, 2007). Recent studies have also demonstrated that serum sTREM-1 levels are increased in inflammatory bowel disease (Schenk et al., 2007), and augmented sTREM-1 levels are found in patients with non-infectious inflammatory conditions such as acute pancreatitis (Yasuda et al., 2008), rheumatoid arthritis (Collins et al., 2009), and chronic obstructive pulmonary disease (Radsak et al., 2007). Thus, sTREM-1 can be considered not only as a diagnostic marker for microbial infections but also as an indicator of disease severity in other inflammatory diseases. Our results clearly showed that treatment with 6-OHDA significantly increased serum levels of sTREM-1 and that LP17 attenuated the 6-OHDA-induced upregulation of sTREM-1. These results are similar to those of a previous study on septic shock (Gibot et al., 2004b). To further applicate our findings from bench to bed, some strategies could be tried to improve the routes of administration.

### Possible Strategies of Improvement in LP17 of Administration Routes of a Potential Clinical Translation

Peptides are generally considered to be worse candidates for potential drugs because of their low oral bioavailability and tendency to be rapidly digested, although most drugs on the market were designed for oral administration. The concept that a drug can be not orally available has become more and more accepted and more pharmaceutical companies have raised interest in peptides as potential drug candidates recently. Compared with chemical drugs, peptide drugs have little side-effects and little drug tolerance, meanwhile, the specificity of treatment is high. However, the peptide drugs need special storage condition, or the activity of the protein may be lost, being otherwise are susceptible to proteolytic enzymes (Sachdeva et al., 2016; Henninot et al., 2018). Another problem is that they can elicit an immune response against themselves within a host by stimulating production of antibodies (Steinstraesser et al., 2009). Therefore, various strategies have been developed to try and overcome the limitations of peptides to increase their *in vivo* plasma residence time. Many cyclic peptides, pseudo-peptides (modification of the peptide bond), and peptidomimetics (non-peptide molecules) preserving the biological properties of peptides have been and are widely developed to increase their resistance to degradation and elimination, their bioavailability, and their selectivity (targeting of protein–receptor interactions) to become good drug candidates (Li and Roller, 2002; Conibear et al., 2016). In fact, from a model peptide of interest (lead peptide), it is often needed to optimize its chemical structure (cyclization, bioisosteric replacement of peptide bonds, changing the stereochemistry of an amino acid) to obtain a compound that can be used therapeutically, even for parental administration (e.g., subcutaneous, intramuscular, or intravenous injection) (Mojsoska and Jenssen, 2015; Qvit et al., 2017). We preliminary examined the possible therapeutic efficacy on PD of modulating TREM-1 activity with LP17. Then, we intend to optimize the chemical structure to suit other methods of administration.

### Linkage Role Calpain-1 Between Neuroinflammation and Neurodegeneration in PD

Calpains are a variety of calcium-dependent proteases which could be able to modulate the function of several target proteins by partial truncation. They regulate several cellular functions through proteolysis, including cytoskeleton assembly and disassembly. Previous studies showed that the activity of calpain significantly improved at supra-physiological levels in a number of neurological disorders [AD or PD (Higuchi et al., 2005; Samantaray et al., 2013)]. In inflammatory process, it is known to be involved in activation of inflammatory cells (T cells, astrocytes, and microglia) and migration of immune cells which also played an important role in PD pathology (Franco and Huttenlocher, 2005). In PD, Levesque et al. (2010) showed that calpain-1 is secreted in the process of dopamine neuron damage and this soluble neuron injury factor is responsible for the toxic aspects of reactive microgliosis. Extracellular calpain-1 could activate microglial NADPH oxidase, producing superoxide to damage neighboring dopaminergic neurons selectively (Levesque et al., 2010). Besides, overlapping characteristics for calpain and caspase-3 has been reported in PD (Burguillos et al., 2011) and motoneuronal cells exposed to Parkinsonian toxins MPP^+^ and rotenone (Samantaray et al., 2011). Hence, we performed the therapeutic effect of LP17 on active caspase-3 in neuroblastoma, SH-SY5Y cell which could confirm its inhibition on calpain-1 in our preliminary study (data not shown).

## Conclusion

**FIGURE 10:**
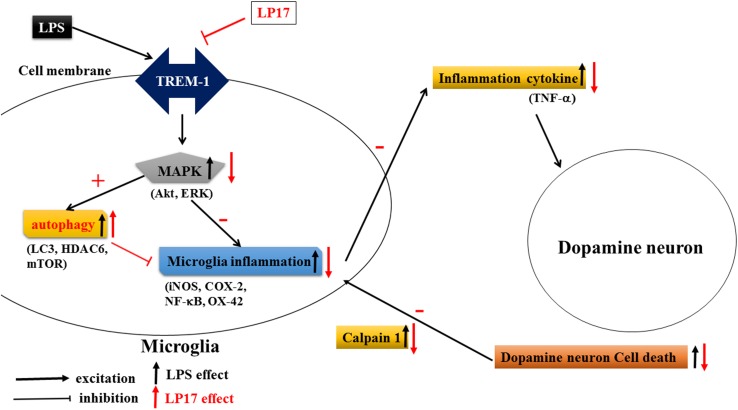
Putative mechanism of action of TREM-1 in LPS-induced neuroinflammation and 6-OHDA-induced neuron cell death. LPS increases the upregulation of TREM-1 protein expression. Downstream MAPK proteins further activate microglial inflammatory processes such as the translocation of NF-κB and upregulation of iNOS and COX-2. Then, the overactivated microglia release various neurotoxic factors such as TNF-α that damage dopamine neurons. These damaged neurons release extracellular calpain-1, further stimulating the microglia and continuing the cycle of neurotoxic microglial activation in response to neuron injury. In our study, the LP17 peptide not only significantly attenuated LPS-induced inflammation process but also enhanced autophagy as indicated by an increase in the LC3-II/LC3-I ratio, upregulation of HDAC6 expression, and downregulation of mTOR. The diminishment of microglial activation may lead to the downregulation of neurotoxic factors and may protect cells against damage. Moreover, LP17 attenuated extracellular calpain-1 release to alleviate the further activation of microglia.

As shown in Figure 10, Inhibition of TREM-1 significantly reduced LPS-induced inflammation and enhanced autophagy. In zebrafish PD model, blockade of TREM-1 attenuated 6-OHDA-induced upregulation of TREM-1 and iNOS and LP17 reversed the 6-OHDA-induced increase in the swimming total distance. We also revealed that LP17 can reverse the increase in the number of rotation behaviors induced by 6-OHDA treatment in rats. Lastly, 6-OHDA-induced attenuation in TH and increase of sTREM-1 were diminished by LP17 treatment in a rat PD model.

## Data Availability

All datasets analyzed for this study are included in the manuscript and the Supplementary Files.

## Ethics Statement

All animal care and experimental use of animals conformed to the Guiding Principles in the Care and Use of Animals of the American Physiology Society and was approved by the National Sun Yat-sen University Animal Care and Use Committee. All studies involving animals are reported in accordance with the ARRIVE guidelines for reporting experiments involving animals. Every effort was made to minimize both the number of animals used and their suffering. Animal use procedures were conducted in strict accordance with the NIH Guide for the Care and Use of Laboratory Animals (8th edition, 2011). Each rat was used only once during the study.

## Author Contributions

C-WF, W-FC, and Z-HW conceived and designed the experiments. N-FC, C-WF, H-CH, and C-SS performed the experiments. C-LC, B-HC, and W-FC analyzed the data. C-WF, N-FC, S-NY, and H-MK contributed to reagents, materials, and analysis tools. W-FC and Z-HW wrote the manuscript.

## Conflict of Interest Statement

The authors declare that the research was conducted in the absence of any commercial or financial relationships that could be construed as a potential conflict of interest.

## Supplementary Material

FIGURE S1 Uncropped Western blots of Figure 1A: TREM-1 and Figure 1B: β-actin.

FIGURE S2 Uncropped Western blots of Figure 2A: iNOS, COX-2, and β-actin.

FIGURE S3 Uncropped Western blots of Figure 2B: IκB, β-actin, NF-κB, and Lamin B.

FIGURE S4 Uncropped Western blots of Figure 3B: LC3, p-mTOR, HDAC-6, mTOR, and β-actin.

FIGURE S5 Uncropped Western blots of Figure 3C: LC3 and β-actin; Figure 3D: p-ERK, ERK, p-Akt, and Akt.

FIGURE S6 Uncropped Western blots of Figure 4A: TREM-1; Figure 4B: iNOS, COX-2, and IκB.

FIGURE S7 Uncropped Western blots of Figure 4C: LC3, HDAC6, and β-actin.

FIGURE S8 Uncropped Western blots of Figure 5: iNOS, COX-2, and β-actin.

FIGURE S9 Uncropped Western blots of Figure 7B: TH, TNF-α, and β-actin.
