# Supplementary figures and images for: Therapeutic Effect of Modulating TREM-1 via Anti-inflammation and Autophagy in Parkinson’s Disease
Source: Front Neurosci. 2019 Aug 2;13:769. doi: 10.3389/fnins.2019.00769 (PMC6691936; doi:10.3389/fnins.2019.00769)

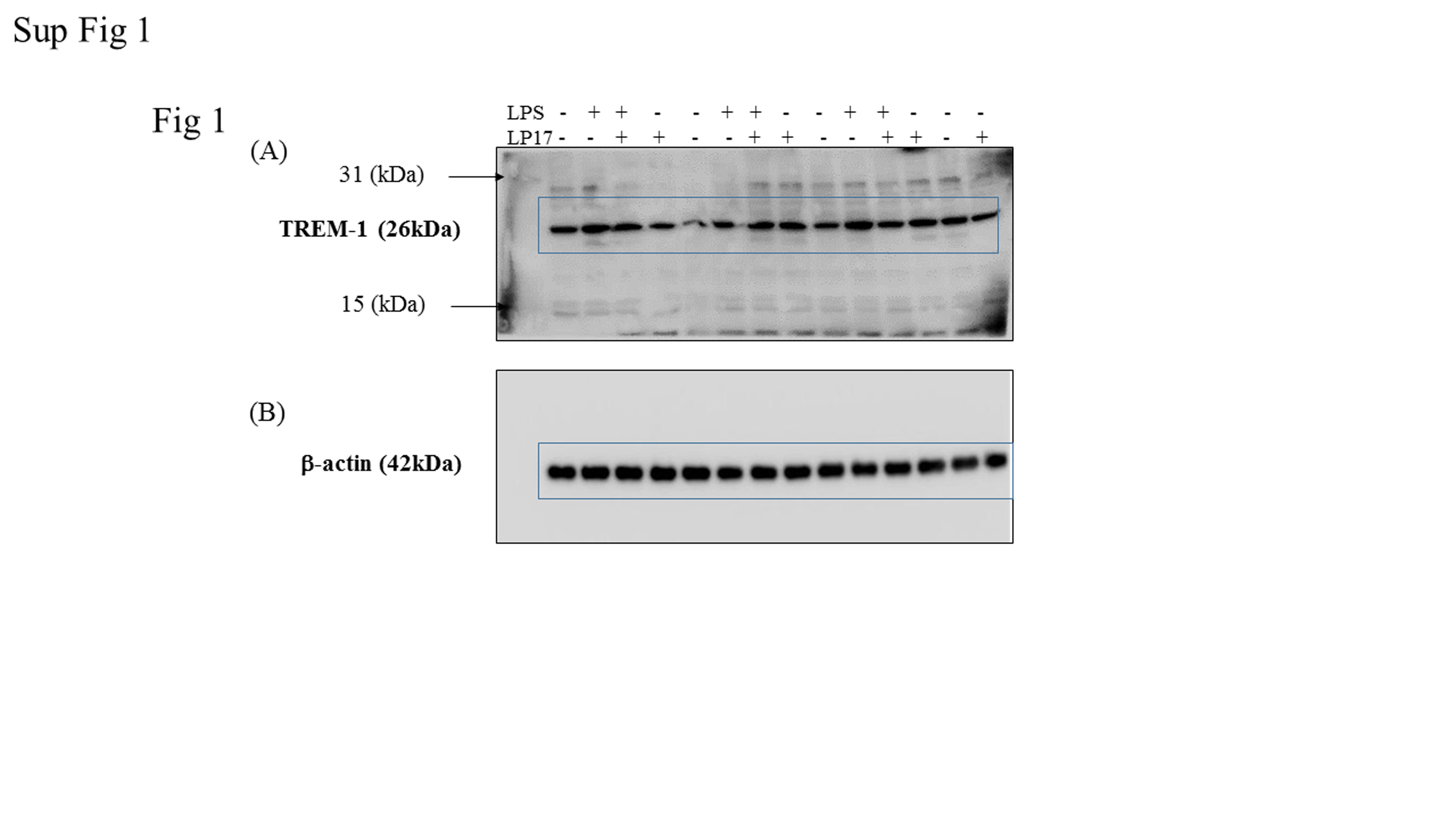

Supplement: FIGURE S1 — Uncropped Western blots of Figure 1A: TREM-1 and Figure 1B: β-actin. [file Image_1.TIF]

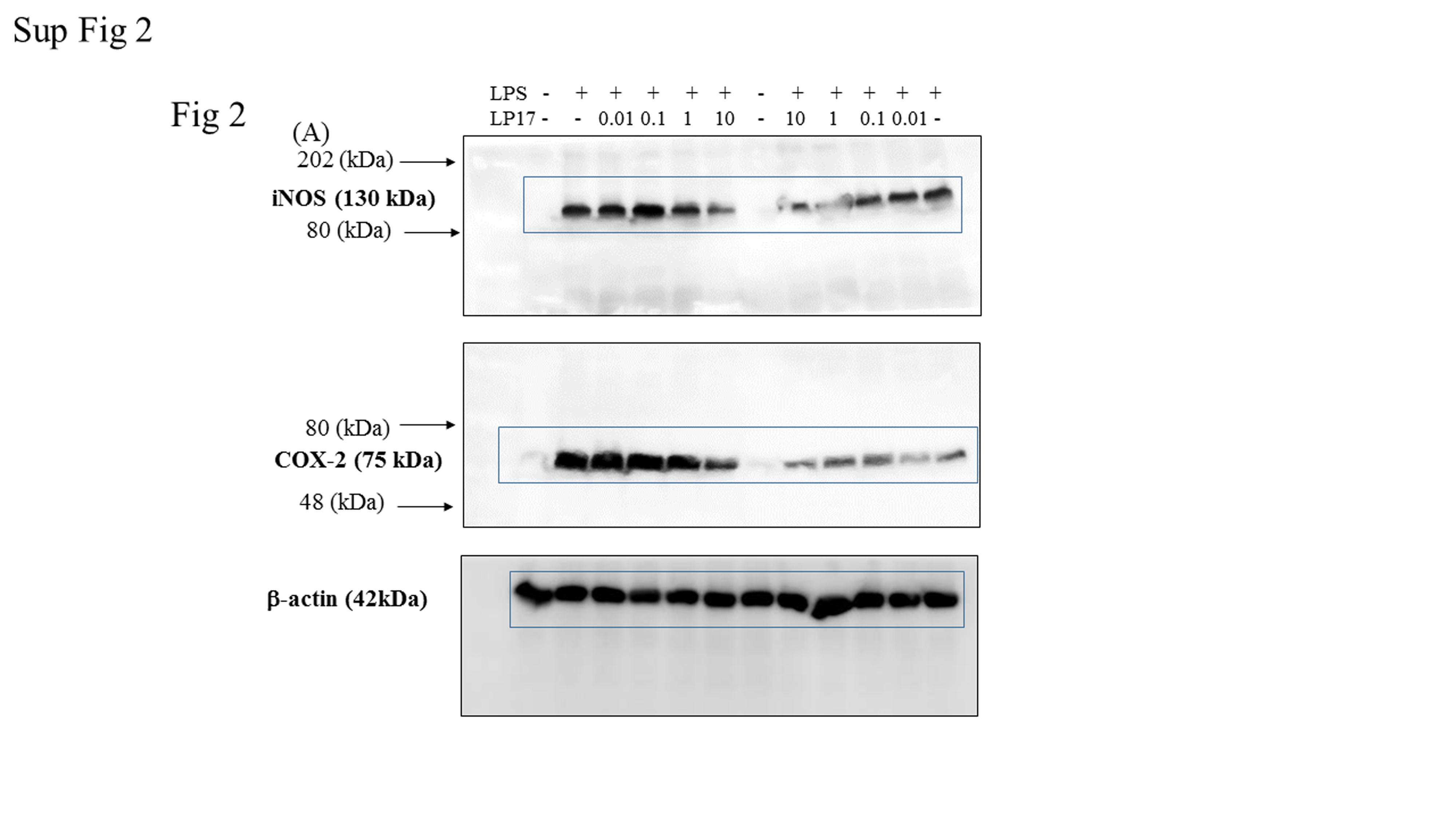

Supplement: FIGURE S2 — Uncropped Western blots of Figure 2A: iNOS, COX-2, and β-actin. [file Image_2.TIF]

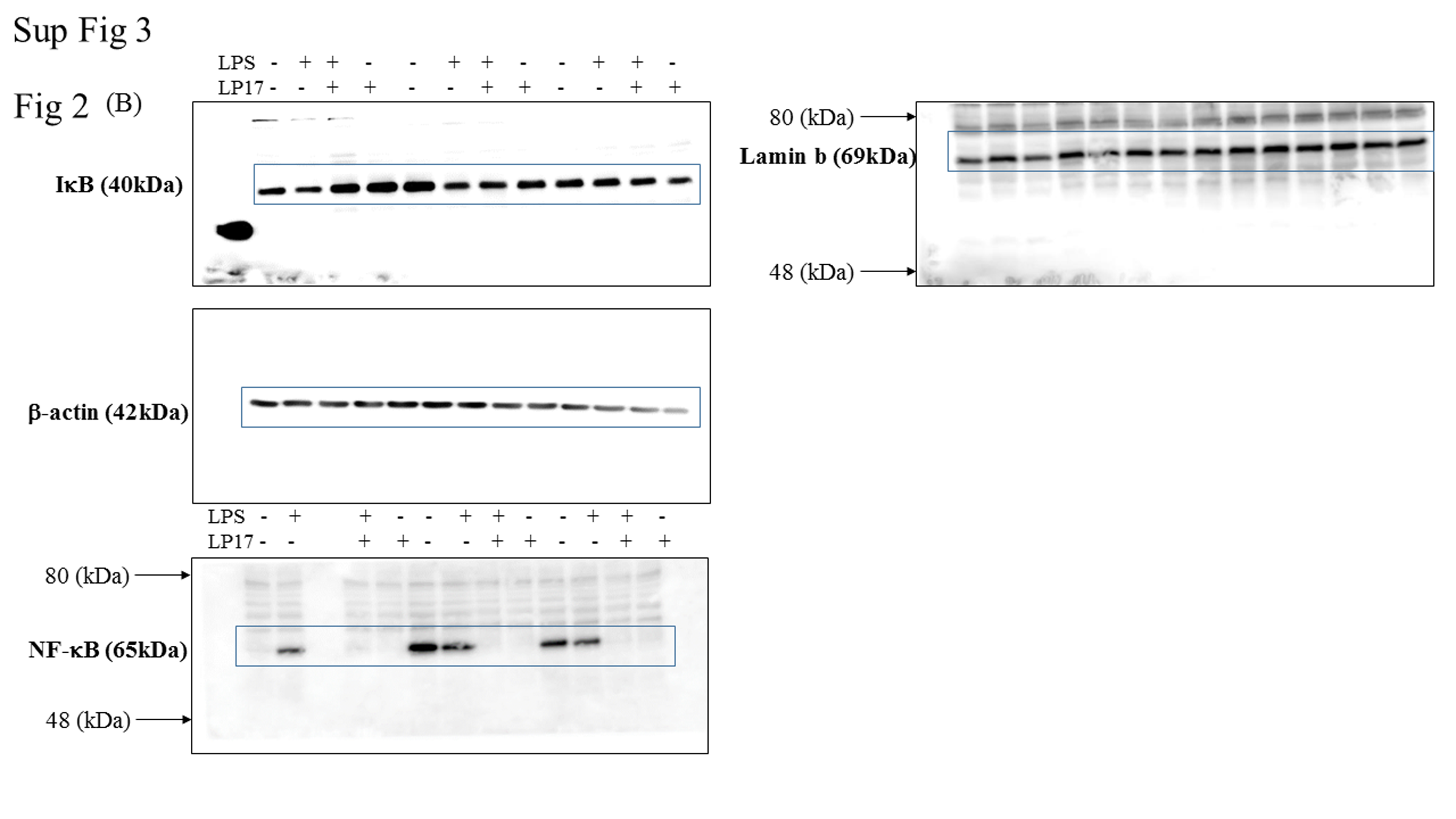

Supplement: FIGURE S3 — Uncropped Western blots of Figure 2B: IκB, β-actin, NF-κB, and Lamin B. [file Image_3.TIF]

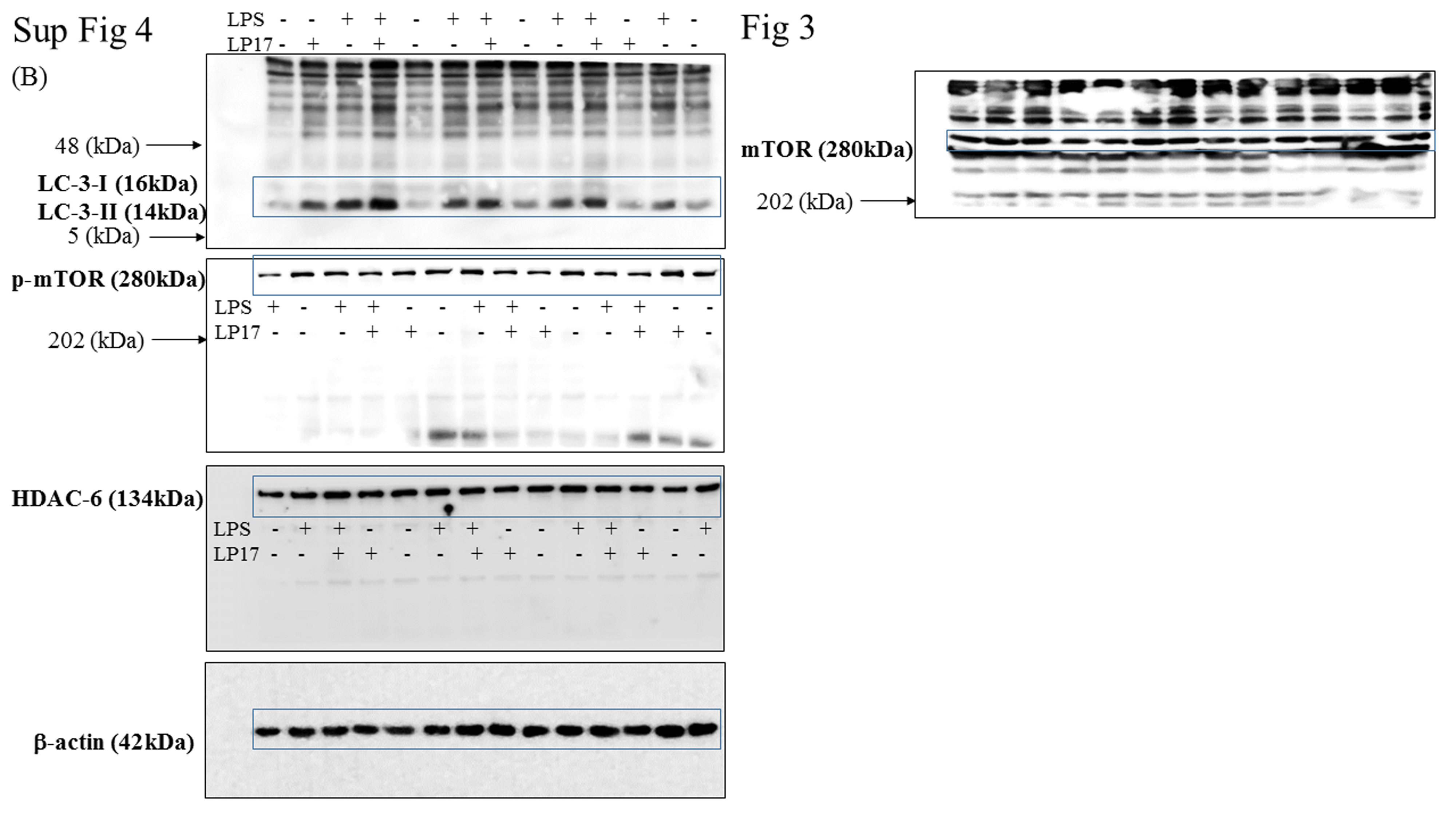

Supplement: FIGURE S4 — Uncropped Western blots of Figure 3B: LC3, p-mTOR, HDAC-6, mTOR, and β-actin. [file Image_4.TIF]

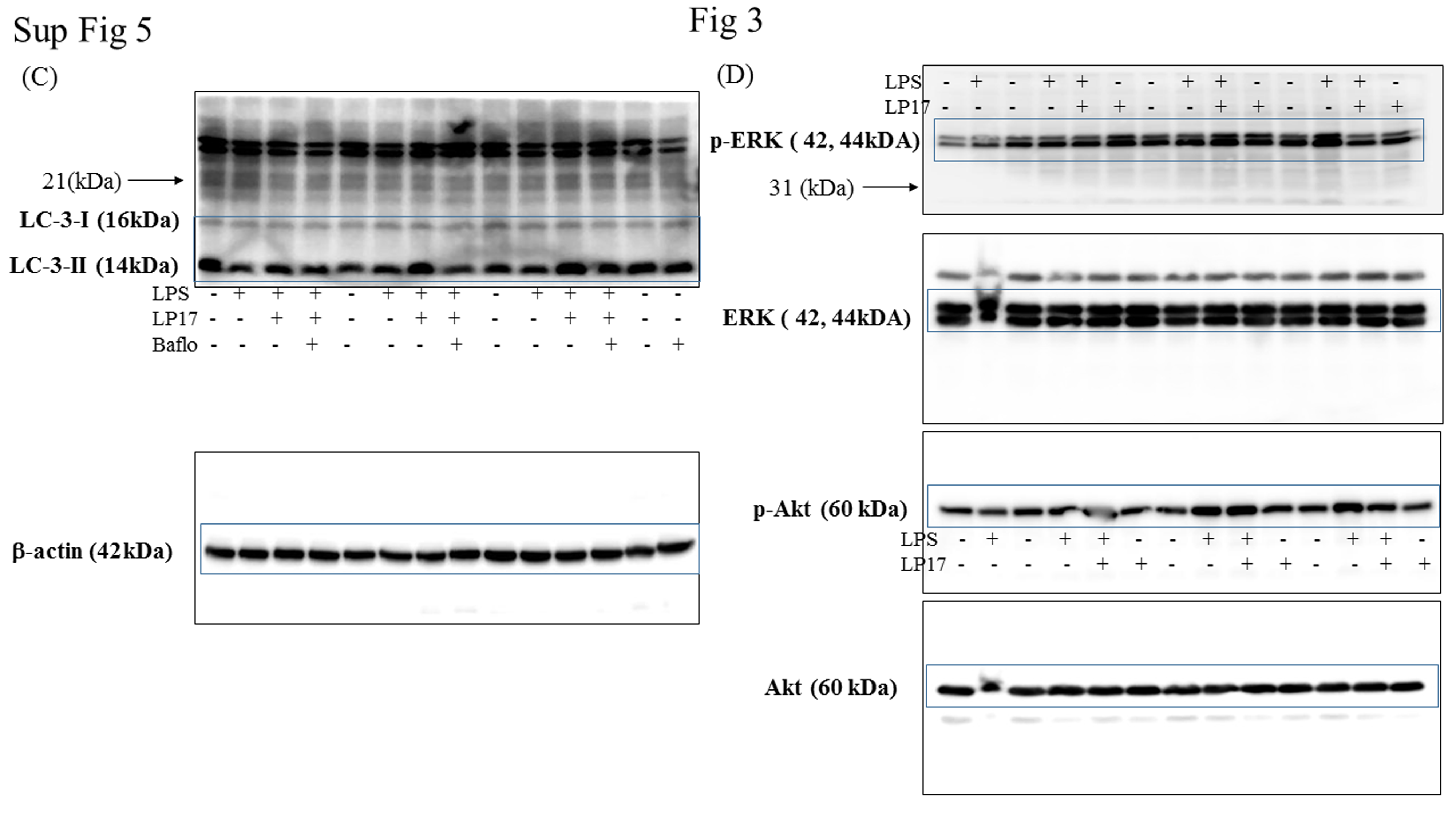

Supplement: FIGURE S5 — Uncropped Western blots of Figure 3C: LC3 and β-actin; Figure 3D: p-ERK, ERK, p-Akt, and Akt. [file Image_5.TIF]

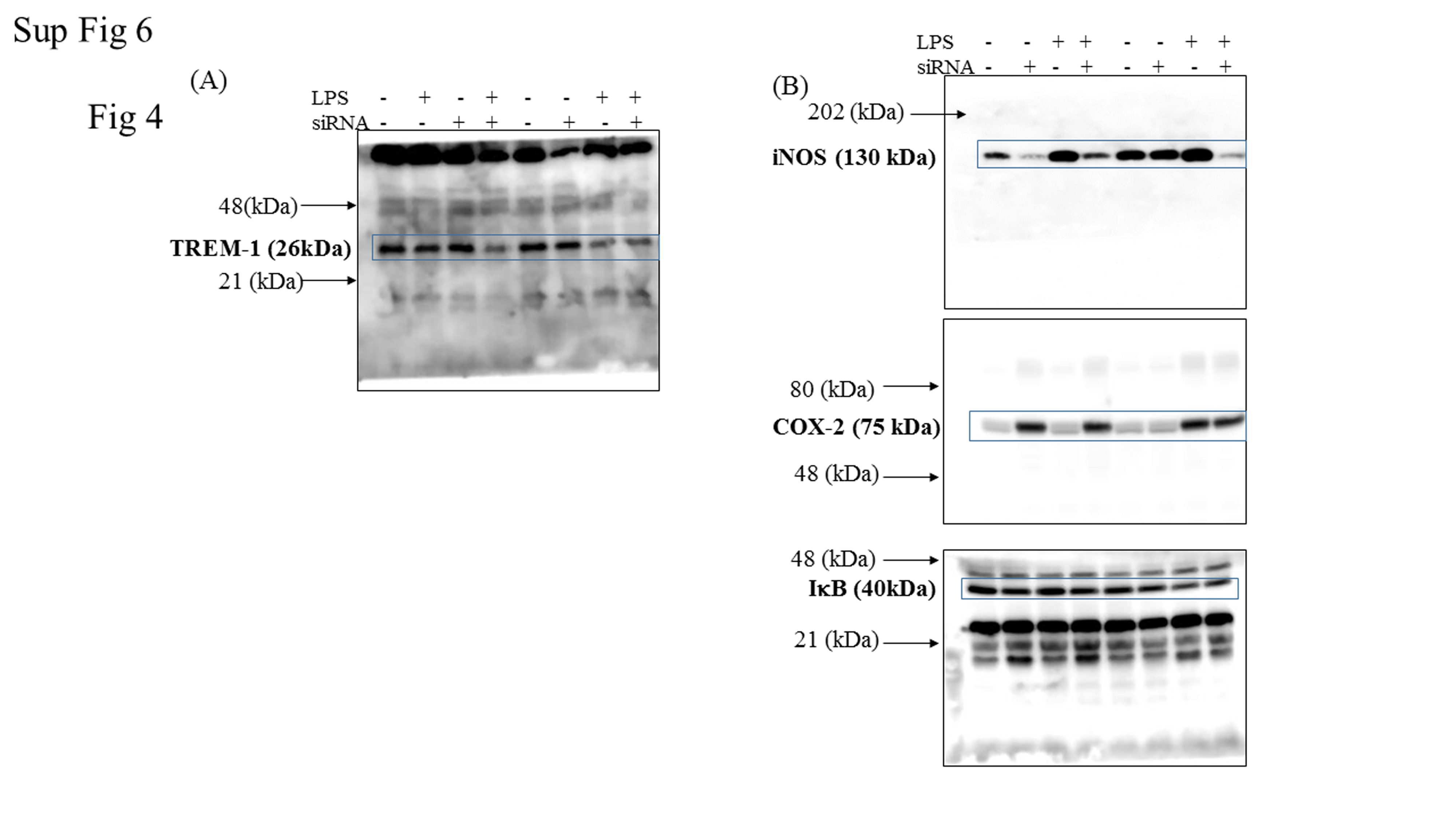

Supplement: FIGURE S6 — Uncropped Western blots of Figure 4A: TREM-1; Figure 4B: iNOS, COX-2, and IκB. [file Image_6.TIF]

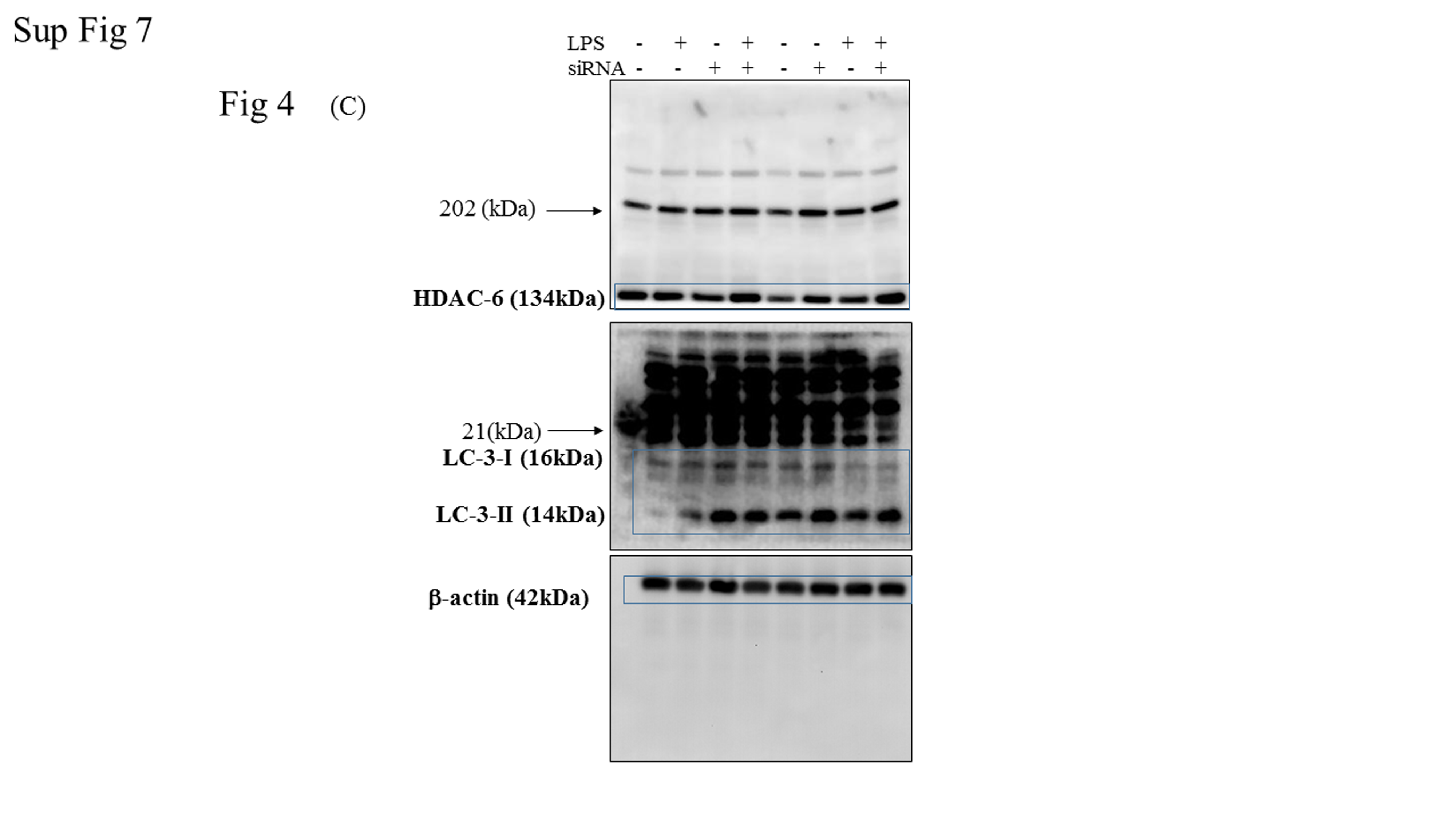

Supplement: FIGURE S7 — Uncropped Western blots of Figure 4C: LC3, HDAC6, and β-actin. [file Image_7.TIF]

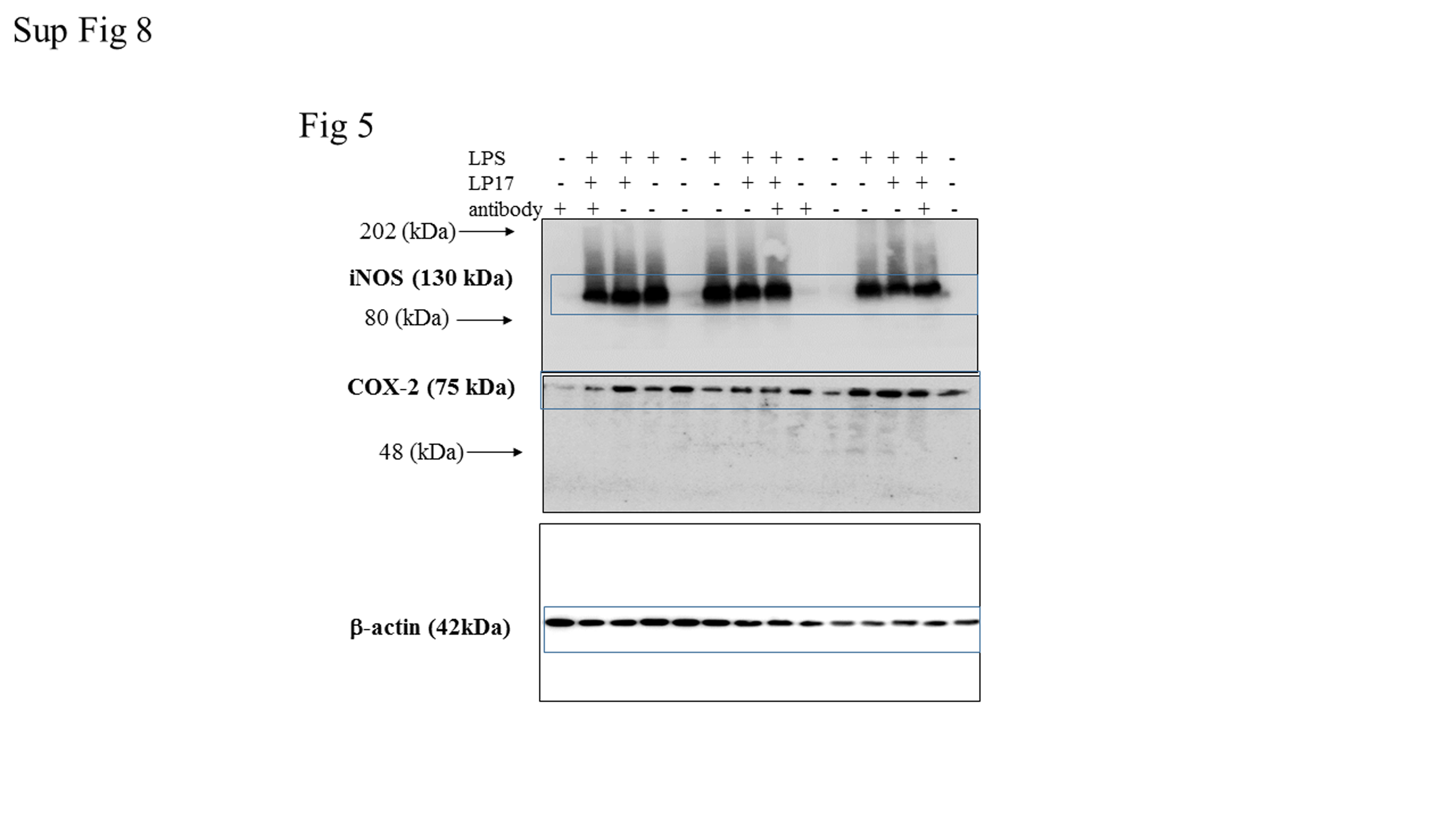

Supplement: FIGURE S8 — Uncropped Western blots of Figure 5: iNOS, COX-2, and β-actin. [file Image_8.TIF]

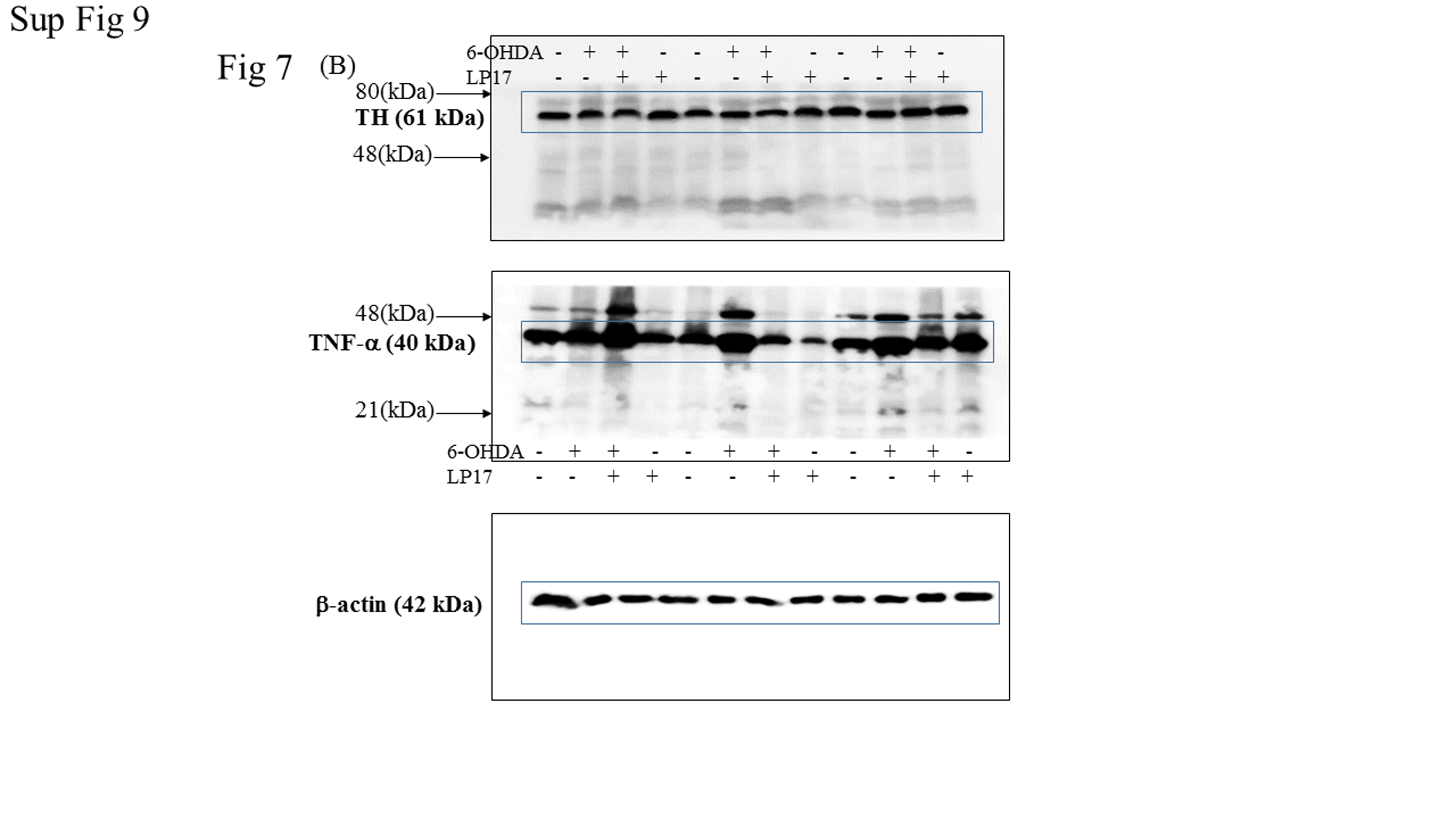

Supplement: FIGURE S9 — Uncropped Western blots of Figure 7B: TH, TNF-α, and β-actin. [file Image_9.TIF]
